# A Primary Care Nurse-Delivered Walking Intervention in Older Adults: PACE (Pedometer Accelerometer Consultation Evaluation)-Lift Cluster Randomised Controlled Trial

**DOI:** 10.1371/journal.pmed.1001783

**Published:** 2015-02-17

**Authors:** Tess Harris, Sally M. Kerry, Christina R. Victor, Ulf Ekelund, Alison Woodcock, Steve Iliffe, Peter H. Whincup, Carole Beighton, Michael Ussher, Elizabeth S. Limb, Lee David, Debbie Brewin, Fredrika Adams, Annabelle Rogers, Derek G. Cook

**Affiliations:** 1 Population Health Research Institute, St George’s University of London, London, United Kingdom; 2 Pragmatic Clinical Trials Unit, Queen Mary’s University of London, London, United Kingdom; 3 College of Health and Life Sciences, Brunel University London, London, United Kingdom; 4 Department of Sport Medicine, Norwegian School of Sport Sciences, Oslo, Norway; 5 MRC Epidemiology Unit, University of Cambridge, Cambridge, United Kingdom; 6 Independent Psychology Research Consultant, Southampton, United Kingdom; 7 Research Department of Primary Care & Population Health, University College, London, United Kingdom; 8 Faculty of Health Social Care and Education, Kingston University and St George’s University of London, London, United Kingdom; 9 10 Minute CBT, Letchworth Garden City, Hertfordshire, United Kingdom; Leiden University Medical Center, NETHERLANDS

## Abstract

**Background:**

Brisk walking in older people can increase step-counts and moderate to vigorous intensity physical activity (MVPA) in ≥10-minute bouts, as advised in World Health Organization guidelines. Previous interventions have reported step-count increases, but not change in objectively measured MVPA in older people. We assessed whether a primary care nurse-delivered complex intervention increased objectively measured step-counts and MVPA.

**Methods and Findings:**

A total of 988 60–75 year olds, able to increase walking and randomly selected from three UK family practices, were invited to participate in a parallel two-arm cluster randomised trial; randomisation was by household. Two-hundred-ninety-eight people from 250 households were randomised between 2011 and 2012; 150 individuals to the intervention group, 148 to the usual care control group. Intervention participants received four primary care nurse physical activity (PA) consultations over 3 months, incorporating behaviour change techniques, pedometer step-count and accelerometer PA intensity feedback, and an individual PA diary and plan. Assessors were not blinded to group status, but statistical analyses were conducted blind. The primary outcome was change in accelerometry assessed average daily step-counts between baseline and 3 months, with change at 12 months a secondary outcome. Other secondary outcomes were change from baseline in time in MVPA weekly in ≥10-minute bouts, accelerometer counts, and counts/minute at 3 months and 12 months. Other outcomes were adverse events, anthropometric measures, mood, and pain. Qualitative evaluations of intervention participants and practice nurses assessed the intervention’s acceptability. At 3 months, eight participants had withdrawn or were lost to follow-up, 280 (94%) individuals provided primary outcome data. At 3 months changes in both average daily step-counts and weekly MVPA in ≥10-minute bouts were significantly higher in the intervention than control group: by 1,037 (95% CI 513–1,560) steps/day and 63 (95% CI 40–87) minutes/week, respectively. At 12 months corresponding differences were 609 (95% CI 104–1,115) steps/day and 40 (95% CI 17–63) minutes/week. Counts and counts/minute showed similar effects to steps and MVPA. Adverse events, anthropometry, mood, and pain were similar in the two groups. Participants and practice nurses found the intervention acceptable and enjoyable.

**Conclusions:**

The PACE-Lift trial increased both step-counts and objectively measured MVPA in ≥10-minute bouts in 60–75 year olds at 3 and 12 months, with no effect on adverse events. To our knowledge, this is the first trial in this age group to demonstrate objective MVPA increases and highlights the value of individualised support incorporating objective PA assessment in a primary care setting.

**Trial Registration:**

Controlled-Trials.com ISRCTN42122561

## Abstract 2

In this cluster randomized controlled trial, Tess Harris and colleagues investigate whether a program delivered by primary care nurses to older adults can increase bouts of moderate to vigorous physical activity.

## Editors’ Summary

**Background:**

Worldwide, people are becoming increasingly sedentary. They are sitting at desks instead of doing manual labor; they are driving to work instead of walking; and they are participating in fewer leisure time physical activities. But the human body needs regular exercise to stay healthy. Physical activity helps to maintain a healthy weight and prevents or delays heart disease, type 2 diabetes, and some cancers. Moreover, physically active people feel better and live longer than physically inactive people. The World Health Organization recommends that adults should be active daily and should do at least 150 minutes of moderate- to vigorous-intensity physical activity (MVPA) every week in bouts that each last at least 10 minutes. Moderate-intensity physical activities (for example, brisk walking) require a moderate amount of effort and noticeably increase the heart rate; vigorous-intensity physical activities (for example, running) require a large amount of effort and cause rapid breathing and a substantial heart rate increase.

**Why Was This Study Done?:**

When physical activity is objectively measured using an accelerometer (a movement monitor that is usually worn on the waist; unlike pedometers, which simply count the number of steps a person takes, accelerometers record both step-counts and the intensity of physical activity), fewer than 5% of older people achieve the recommended weekly levels of MVPA. These levels could be reached by taking regular brisk walks but how can older people be persuaded to make such walks part of their daily lives? In this cluster randomized controlled trial—the PACE (Pedometer Accelerometer Consultation Evaluation)-Lift trial—the researchers assess whether an intervention to increase walking comprising pedometer and accelerometer feedback combined with physical activity consultations provided by practice nurses can lead to sustained increases in physical activity in 60–75 year olds. Cluster randomized trials compare outcomes in groups of people (here, husbands and wives living in one household) assigned through the play of chance to receive a test or a control intervention.

**What Did the Researchers Do and Find?:**

The researchers assigned 298 people from 250 households to receive the intervention or usual care and determined the participants’ physical activity at baseline, 3 months, and 12 months by asking them to wear an accelerometer for 7 days. Intervention group participants received four physical activity consultations with a primary care nurse over 3 months. At the first consultation, the nurse gave each participant a pedometer to measure step-counts, a physical activity diary in which to record their goals and progress, and the PACE-Lift patient handbook. At each consultation, the nurse used behavior change techniques (for example, goal setting) to encourage the participant to walk regularly, gave the participant visual feedback from their accelerometer readings, and devised a personal walking plan. Control group participants received normal care only from the practice. At 3 months and 12 months, both the average daily step-count and the weekly MVPA level had decreased from baseline in the control group but increased in the intervention group. At 3 months, compared to the control group, the average step-count in the intervention group was 1,037 steps/day higher and the MVPA in bouts of more than 10 minutes in the intervention group was 63 minutes/week higher. At 12 months, the corresponding differences were 609 steps/day and 40 minutes/week. There were no significant differences in adverse events (for example, pain), body fat, or other measured health-related outcomes between the groups.

**What Do These Findings Mean?:**

These findings show that the complex intervention tested in the PACE-Lift trial increased both step-counts and objectively measured MPVA among 60–75 year olds for at least 12 months. Importantly, all the participants and practice nurses were positive and enthusiastic about the intervention. Moreover, the observed increase in physical activity is estimated to reduce the participants’ risk of heart disease and type 2 diabetes by an estimated 5.5% and 9.1%, respectively. Further trials are needed to determine which aspects of the intervention were responsible for increasing physical activity, to determine whether the intervention’s effects will persist for longer than 12 months, and to test the intervention in socio-economically diverse populations (all the PACE-Lift participants came from an affluent, non-ethnically diverse area). However, these findings highlight the value of family practices providing older individuals with individualized support that incorporates objective assessment of physical activity to help them become more physically active.

**Additional Information:**

Please access these websites via the online version of this summary at http://dx.doi.org/10.1371/journal.pmed.1001783.

- The US Centers for Disease Control and Prevention provides information on all aspects of healthy living; its Physical Activity for Everyone web pages include guidelines for older people, instructional videos, and personal success stories (some information in English and Spanish)
- The World Health Organization provides information about physical activity and health (in several languages); its Global Recommendations on Physical Activity for Health are available in several languages
- The UK National Health Service information source Choices explains the benefits of regular physical activity, provides recommendations for older people, and includes tips for exercising and some personal stories
- MedlinePlus has links to other resources about exercise and physical fitness (in English and Spanish)
- More information about the PACE-Lift trial protocol is available

## Introduction

Physical activity (PA) is an important determinant of health and well-being in older people and reduces mortality [1]. Older adults are advised to be active daily and to accumulate at least 150 minutes of moderate to vigorous intensity PA (MVPA) weekly, in at least 10 minute bouts. This can be achieved through regular walking [1,2]. Recent surveys based on objective PA assessment suggest that less than 5% of this age group achieve recommended PA levels [3,4].

Increasing older people’s PA is challenging. Behaviour change techniques (BCTs) (e.g., goal-setting, self-monitoring, building self-efficacy) are more effective than health education alone [5]. Best practice includes gradually increasing to moderate intensity, incorporating PA into daily routines (e.g., walking) and monitoring intensity [5]. Interventions tailored to people’s needs and delivered at the individual or household level can encourage walking [6]. Primary care provides an ideal context for PA interventions; it allows population-based sampling of healthy older people (by individual or household), practice nurse involvement, and continuity of care, with many chronic diseases being indicators for increasing PA. United Kingdom National Health Service (NHS) Health Checks include adults to age 74 and incorporate brief advice on increasing PA, often by practice nurses or health care assistants [7].

Pedometers provide direct step-count feedback; accelerometers need computer analysis, but record step-counts and PA intensity. Three systematic reviews found pedometer users increased steps/day by 2,000–2,500, but trials included few older adults and focused on volunteers or patients with specific conditions, mainly with short follow-up (3 to 6 months) [8–10]. Two more recent older adult population-based primary care trials showed significant step-count increases at 3 [11,12] and 6 months [12], but did not report on MVPA and included few men [11,12].

We therefore conducted a randomised trial to determine whether an intervention based on pedometer and accelerometer feedback combined with practice nurse PA consultations increases PA levels in 60–75 year olds over 3 months and whether any change is maintained at 12 months. Secondary aims were to assess whether effects were modified by age, gender, body mass index (BMI), disability, exercise self-efficacy or taking part as a couple, and to estimate effects on patient reported outcomes and anthropometric measures. The trial was designed to be population-based and to measure objectively assessed PA, including MVPA (providing a direct link to PA guidelines). Qualitative studies were undertaken with intervention participants and practice nurses to assess the intervention’s acceptability and the barriers and facilitators to increasing PA.

## Methods

### Trial Design

The trial protocol has been published (S1 Text) [13]. A two-arm parallel cluster randomised trial, randomised by household, compared a complex intervention to increase walking carried out over a 3 month period with a usual care control group. The primary endpoint was assessed at 3 months. Further follow-up assessed maintenance of any differences between groups at 12 months.

### Ethical Review

The trial was approved by Oxfordshire Research Ethics Committee C, UK (11/H0606/2).

### Participants

Eligible participants were patients aged 60–74 years registered at three general practices in Oxfordshire and Berkshire, UK, who could walk outside and had no contra-indications to increasing PA. Patients were excluded if they were in a residential or nursing home or were identified as having a medical or psychiatric condition unsuitable for the intervention either through Read code from their electronic primary care records or by their general practitioner (family practice doctor) prior to invitation mail-out (S1 Text) [13]. A random sample of 200 eligible households, with either one or two older adults at the same address, was selected per practice. Individuals in these households were mailed an invitation and a reminder if no response was received after 6 weeks. Further households were randomly selected until 100 individuals per practice were randomised.

### Baseline Assessment Procedures

Interested patients attended a baseline assessment at the practice, where individual informed written consent was obtained. Two eligible people in a household were invited together (apart if preferred). Recruitment was between October 2011 and September 2012. The baseline questionnaire, which assessed a range of health and lifestyle factors, was administered, and height, weight, and fat mass measured (S1 Text) [13]. Participants were asked to continue with their usual PA while wearing a hip-mounted accelerometer (GT3X+, Actigraph LLC) to record PA (in 5 s epochs) all day (between rising and going to bed) for 7 consecutive days, except for bathing, and to keep a contemporaneous PA diary. Accelerometers and diaries were returned to the practice. Participants not providing ≥5 days of ≥540 minutes (9 hours) per day of data were asked to rewear the accelerometer.

### Randomisation and Masking

Participants with adequate baseline accelerometer data were randomised using the Nottingham Clinical Trials Unit internet randomisation service. Randomisation was at household level to avoid couple contamination. Block randomisation was used within practice with random sized blocks, varying between 4 and 6, and 1:1 allocation ratio, to ensure group balance and an even nurse workload. Participants were informed by telephone of their group allocation. Researchers were not blind to intervention status at assessments.

### Procedure for the Control Group

Control group participants received usual care from the general practice, with no trial contacts other than for data collection at baseline, 3 months, and 12 months.

### Procedure for the Intervention Group

The researcher arranged a first nurse appointment for participants (either as an individual or as a couple). The practice nurse arranged the three further appointments for participants, which were 2, 6, and 10 weeks later. The main focus of the intervention was the four individually tailored PA consultations with the practice nurse. Data collection for trial outcomes was as for the control group at 3 months and 12 months.

### Complex Intervention Components

The components of the intervention are summarised in Table 1 and have been described previously (S1 Text) [13]. The key components were as follows: (i) pedometers (SW-200, Yamax Digi-Walker) given to participants by nurses at their first visit to record their step-counts in the PA diary; (ii) accelerometers (GT3X+, Actigraph) that participants were asked to wear before the second, third, and fourth nurse visits and from which nurses downloaded data during the consultation to show participants the time they were spending in different PA intensities; (iii) practice nurse consultations based on key BCTs including goal-setting, self-monitoring, building self-efficacy and social support, overcoming barriers, preventing relapses, and building lasting habits; (iv) PACE-Lift patient handbook, which supported BCTs, given to patients to keep at their first appointment and used during consultations; (v) individual walking/PA plan agreed upon during consultations between nurses and participants to encourge adding in both steps and time spent walking in moderate intensity PA, in bouts of at least 10 minutes, to each individual’s baseline; (vi) PA diary to record PA and step-counts, used with the monitors to set goals, monitor progress, and aid feedback, by relating specific diary activities to accelerometer recorded PA intensities.

**Table 1:** Components of the complex intervention for the PACE-Lift trial.

Components	What Was Provided	Additional Detail on Components
Pedometer	Yamax Digi-Walker SW-200 model	Nurse gave to patient at first visit with instructions for use. Provided direct step-count to participants and required daily manual recording and re-setting. Asked participants to wear for 7 days before subsequent nurse visits and to record step-counts in PA diary. Could wear more often if they wished.
Accelerometer	Actigraph GT3X+ (LLC)	Asked participants to wear for 7 days before second, third, and fourth nurse visits. Recorded time spent in different PA intensities (sedentary, light, moderate, vigorous) [13]. The nurse downloaded data to a computer and provided visual feedback to participants at each visit.
Practice nurse PA consultations	Four individually tailored PA consultations with the practice nurse. Participants could be seen individually or as a couple.	PA consultations with a practice nurse based on BCTs. Session timing, content, and proposed BCTs are fully described elsewhere [13]. Pedometers, accelerometers, walking plan, and step-count diaries were planned as useful adjuncts to BCTs.
Patient handbook	Patient handbook to support 12-week walking programme	PACE-Lift patient handbook, including BCTs, adapted from the NHS Health Trainer Handbook but focusing only on PA behaviour change, was produced for the trial, used for nurse training and by nurses during their consultations and given to individual patients to keep at their first appointment. (See protocol for details [13].)
Walking/PA plan	Individual walking/PA plan	An individual walking /PA plan, devised during nurse PA consultations. We did not use a single goal (e.g., 10,000 steps/day) but instead nurses encouraged steps and time spent walking in moderate intensity PA, particularly in bouts of at least 10 minutes, to be added incrementally to each individual’s baseline. Nurses also encouraged discussion of when participants would walk, where, and who with.
PA diary	PA diary to record weekly PA for 12 weeks (step-count and walks)	A diary to record PA and step-counts, used with the monitors, to set goals, monitor progress, and aid feedback by relating specific diary activities to accelerometer recorded PA intensities.

The PACE-Lift patient handbook and PA diary are available in S3 and S4 Texts. Practice nurse training, supervision, support, and methods for ensuring intervention fidelity have been described previously (S1 Text) [13].

### 3 Month Assessment and Outcome Measures

The researcher arranged a 3 month outcome assessment, conducted as per baseline, at the participant’s practice, including wearing the accelerometer for a further 7 days, for both groups. The primary outcome was change in average daily step-count between baseline and 3 months. The secondary outcomes were changes in average weekly time spent in MVPA, MVPA in >10 minute bouts, and in accelerometer counts and counts/minute of wear-time between baseline and 3 months. All primary and secondary outcomes were assessed by accelerometry. Ancillary outcomes were changes in depression (15-item Geriatric Depression Score), anxiety (4-item FEAR score), and pain (4-item self-reported pain score) (see protocol [S1 Text] for full references for all questionnaire measures [13]), BMI, and percentage body fat. Adverse events were collated from contemporaneous spontaneous patient reports to nurses or researchers for trial safety purposes. Adverse events were also collected retrospectively from questionnaires in a systematic manner for control and intervention participants at 3 months for trial outcome reporting. Questions covered falls inside and outside the house, fractures, sprains or injuries, and deterioration in health problems.

### 12 Month Assessment

The 12 month assessment was based on postal accelerometry and questionnaires, similar to those for the 3 month assessment. No anthropometric measurements were made.

### Data Reduction

ActiGraph data were reduced using Actilife software (v6.6.0) set to ignore runs of ≥60 minutes of zero counts [4,13]. We used axis 1 counts as these are the basis of the validated step and MVPA algorithms. The analysis summary variables were: steps; accelerometer wear-time; total daily counts, counts per minute of wear-time (CPM); time spent in MVPA (≥1,952 CPM, ≥3 METs) using standard Freedson cut-points [14]; and time spent in ≥10-min bouts of MVPA. Accelerometer counts are a dimensionless number that quantify the amplitude and frequency of the acceleration signal; they are brand specific.

### Changes from Planned Analysis in Protocol

**Fig 1:**
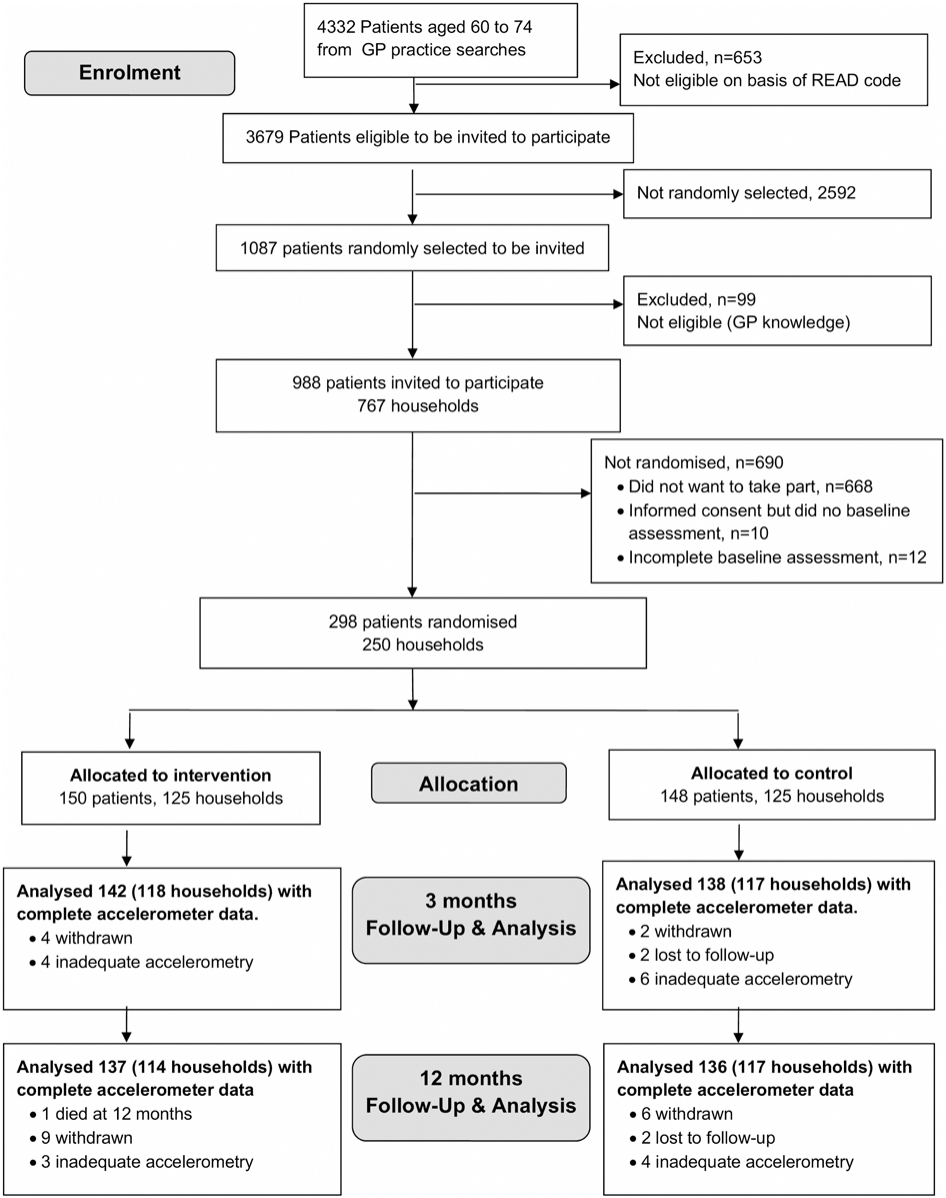
PACE-Lift CONSORT diagram.

Patients were aged 60–74 years when they were selected; however, some were aged 75 at randomisation, therefore the primary aim now relates to 60–75 year olds. The protocol stated that patients should have ≥5 days of ≥600 minutes recorded activity to be randomised; 24 patients were randomised not meeting this criteria. The criteria for “adequate” wear-time varies, but researchers generally use criteria of a minimum of 3–5 days of data [15], with either 600 [16] or 480 [17] minutes/day of data. Prior to data unblinding, and agreed by the Trial Steering Committee, the criteria was relaxed to allow days of ≥540 minutes of data to be included, minimising missing days, whilst maximising the likelihood that the data included in analyses accurately reflected activity for the day measured. Thus patients were considered correctly randomised if they had ≥5 days with ≥540 minutes of data/day. Twelve people were incorrectly randomised (i.e., with <540 minutes data on 5 days) (Fig. 1) as incomplete baseline assessment. At 3 and 12 months, analysis includes any day with ≥540 minutes, to lessen attrition bias.

### Statistical Power and Analyses

A sample of 300 individuals was required to detect a difference of 850 steps/day at 3 months between the two groups with 90% power, 1% significance, and allowing for household clustering and 10% attrition [13]. Analysis and reporting followed CONSORT guidelines [18]. Statistical analyses were carried out blind to study group.

### Analysis of Outcomes

Our primary analysis was based on analyzing all participants with ≥1 day of 540 minutes wear-time at 3 months. We fitted a multi-level model in STATA using the xtmixed procedure. Level 1 was day within individual, level 2 was individual, and level 3 household. The multi-level model used assumed the random effects were independent and the model was fitted using maximum likelihood. Number of steps on a given day at 3 months was regressed on day order of wear, day of week, month of baseline accelerometery, age, gender, practice, treatment group, and estimated average daily steps at baseline. By including baseline daily steps as a covariate, this effectively measured change in step-count over the 3 months, minimising bias and maintaining power [19,20]. Estimated average daily steps at baseline was derived by using a similar two-level model in which daily steps were regressed on day order of wear and day of week. Secondary outcome measures were number of minutes spent in MVPA and MVPA in bouts of ≥10 minutes, as well as counts and counts per minute. These measures were highly correlated with step-count and were analysed using identical approaches. In all presentations MVPA is expressed as estimated number of minutes spent in MVPA (or MVPA in ≥10-minute bouts) per week. Checks were carried out to confirm that the distribution of residuals from the regression models for change in steps and MVPA were normally distributed (S1 and S2 Figs.).

### Ancillary Variables

The effects of the intervention on change in depression, anxiety and pain scores, BMI, and body fat were estimated using a two-level model in which outcome was regressed on the same measure at baseline, practice, month of baseline treatment, age, gender, and treatment. Level 1 was individual, level 2 household.

### Adverse Events

Numbers who suffered adverse events between 0 and 3 months (spontaneously reported or systematically collected from the 3 month questionnaire) were compared between groups using Fisher’s exact test.

### Subgroup Analyses

For the primary outcome only, interaction terms were added to the regression model to test whether the intervention effect varied between: men and women; different age groups; different BMI cut-offs; different disability levels; different levels of confidence in ability to exercise; and those taking part as a couple versus those taking part individually.

### Sensitivity Analyses

The primary analysis, while statistically efficient, gives greater weight to participants recording more valid days of accelerometry at 3 months. To assess the effect of this, average daily step-counts at 3 months were estimated in the same way as average baseline steps. The 3 month average daily step-count was regressed on baseline average daily step-count in a two-level model as for other outcome variables to assess the treatment effect, thus giving equal weight to each participant in the analysis. To assess if participants lost to follow-up, or who failed to record a single adequate day at 3 months, might have introduced bias, we used the STATA procedure *mi impute* to impute average steps/day at 3 months based on baseline steps, gender, age, month of baseline accelerometry, practice, and treatment group. Further analyses added postcode derived Index of Multiple Deprivation (IMD) score [21] as national quintiles, self-reported pain, and fat mass to the imputation. Sensitivity analyses also explored the possible impact of outcomes not being missing at random. We assumed that control participants with missing data experienced no change. In the treatment group we explored scenarios of no change and ±3,000 steps at 3 months and ±1,500 steps at 12 months. Finally, sensitivity analyses were performed repeating the main analyses using the original criteria of ≥600 minutes/day wear-time.

We repeated the primary analyses and assessment of adverse events at 12 months.

### Qualitative Evaluations with Intervention Participants and Practice Nurses

We examined the experiences of participants who did and did not increase their walking in order to determine the acceptability of the intervention and the barriers and facilitators to PA for trial participants. An experienced qualitative interviewer (AR) conducted 30 semi-structured telephone interviews, using a topic guide, with a purposive sample of 19 participants who had increased their walking at 3 months and maintained this at 12 months (improvers) and 11 participants who had not (non-improvers). Interviews were audio-recorded, transcribed, and coded independently by researchers to generate a thematic coding framework.

We explored the practice nurses’ evaluation of the acceptability of the intervention in a group interview with four practice nurses who had delivered the intervention (two at one practice, one at each of the other practices) facilitated by experienced qualitative researchers (CRV, AW) using a semi-structured interview guide. The interview was audio-recorded, transcribed, and coded independently.

## Results

### Participants

Of 988 patients invited, 298 (30%) were randomised to intervention (*n* = 150) or control (*n* = 148) groups (Fig. 1). Six participants withdrew and two were lost to follow-up (unable to be contacted) at 3 months (8/298, 3%). A total of 280/298 (94%) provided accelerometer data with at least 1 day of ≥540 minutes wear-time and were included in the primary outcome analysis at 3 months. Of these, 135/138 (98%) patients in the control group and 140/142 (99%) individuals in the intervention group provided ≥3 days of ≥540 minutes wear-time data (S1 Table). At 12 months, 15 patients had withdrawn, two were lost to follow-up, and one had died (18/298, 6.0%). Baseline characteristics were similar between randomised groups except that participants in the intervention group were on average slightly older than those in the control group, more likely to have left school younger, be overweight or obese, more likely to have chronic diseases and disability, and a slightly lower baseline step-count (Table 2). Over 70% of both groups (104/148 parcipants in control group and 114/150 parcipants in intervention group) were from the least deprived national quintile of Index of Multiple Deprivation (IMD) [21] Accelerometer wear-time was similar in the control and intervention groups at baseline (Table 2).

**Table 2:** Baseline characteristics of 298 randomised participants.

Characteristic	Control Group, *n* = 148		Intervention Group, *n* = 150	
	n	(%)	n	(%)
**Age at randomisation**				
60–64 years	69	(47)	41	(27)
65–69 years	44	(30)	61	(41)
70–75 years	35	(24)	48	(32)
**Gender:** male	69	(47)	69	(46)
**Marital status**				
Married	117	(80)	123	(82)
Widowed	9	(6)	12	(8)
Divorced or separated	14	(10)	10	(7)
Single	7	(5)	5	(3)
**Retired**	80	(55)	95	(63)
**Ethnicity:** white	143	(98)	147	(99)
**Age finished full-time education**				
≤16	54	(37)	67	(46)
17–18	20	(14)	25	(17)
19+	71	(49)	55	(37)
**National quintiles of Index of Multiple Deprivation Rank^a^**				
1–3 (most deprived)	16	(11)	13	(9)
4	28	(19)	23	(15)
5 (least deprived)	104	(70)	114	(76)
**Current smoker**	10	(7)	6	(4)
**General health^b^:** very good or good	133	(92)	127	(86)
**Chronic diseases^b^**				
None	52	(35)	39	(26)
1–2	84	(57)	94	(63)
≥3	12	(8)	17	(11)
**Presence of self-reported pain^b^**	100	(68)	101	(68)
**Limiting long-standing illness^b^**	32	(23)	40	(28)
**FRAT^b^ (0–5)**				
Low risk, score 0	92	(64)	98	(66)
Medium risk, score 1–2	48	(33)	44	(30)
High risk, score 3+	4	(3)	6	(4)
**Geriatric Depression Score^b^** (0–15): high (≥5)	11	(8)	7	(5)
**Fear anxiety score^b^ (0–4):** high (2–4)	22	(15)	22	(15)
**Townsend disability score^b^**				
None (0)	111	(76)	93	(63)
Mild disability (1–5)	31	(21)	51	(35)
Moderate or severe disability (6–18)	4	(3)	3	(2)
**Low self-efficacy score^b^**	34	(24)	37	(26)
**Receiving occupational pension**	105	(74)	109	(77)
**Difficulty in paying bills^b^:** ever	15	(10)	17	(12)
**Randomised as a couple**	47	(32)	52	(35)
**Season of baseline measure**				
March–May	40	(27)	29	(19)
June–August	39	(26)	48	(32)
September–November	38	(26)	41	(27)
December–February	31	(21)	32	(21)
**Physical characteristics**				
**BMI:** overweight/obese (≥25kg/m^2^)	92	(62)	108	(72)
	Mean	(SD)	Mean	(SD)
**Fat mass^c^ (kg)**	23.2	(9.7)	24.5	(8.5)
**Waist circumference^c^ (cm)**	95.2	(14.1)	96.5	(11.2)
**Accelerometry data^c^**				
**Adjusted baseline step count per day**	7,380	(2,988)	7,314	(2,693)
**MVPA**				
Total weekly minutes	301	(169)	296	(154)
Total weekly minutes in ≥10 minute bouts	88	(113)	96	(104)
**Adjusted baseline counts per day**	246,610	(111,809)	244,225	(94,980)
**Adjusted baseline counts per minute of wear-time**	310	(129)	306	(112)
**Adjusted average daily wear-time (minutes)**	789	(72)	797	(79)

^a^National quintiles of Index of Multiple Deprivation rank [21].

^b^Full references for general health, chronic disease score, limiting long-standing illness, self-reported pain, Falls Risk Assessment Tool (FRAT), Geriatric Depression Score (GDS-15), FEAR anxiety score, Townsend Disability Score, Self-Efficacy Score, difficulty in paying bills, are given in the trial protocol [13].

^c^For fat mass, waist circumference, and all accelerometry data, numbers presented are mean (standard deviation [SD]).

Among intervention participants, 129/150 (86%) attended all four nurse sessions. Participants were asked to wear an accelerometer for the week before sessions 2–4; this occurred before 98% (415/425) of nurse sessions attended.

**Effect of the intervention on PA at 3 and 12 months.** Average daily step-count increased at 3 months in the intervention group and decreased in the control group; the between-group difference in change was 1,037 (95% CI 513–1,560) steps/day (*p* < 0.001) (Table 3). All coefficients from the model fitted are given in S2 Table. Time spent in MVPA followed the same pattern; the between-group difference was 66 (95% CI 36–96) minutes/week (*p* < 0.001). Of this number, the difference in ≥10 minute bouts of MVPA was 63 (95% CI 40–87) minutes/week (*p* < 0.001). At 12 months the corresponding differences were 609 (95% CI 104–1,115) steps/day (*p* = 0.018) and 40 (95% CI 10–70) minutes/week MVPA (*p* = 0.009); all of the increased MVPA was in ≥10 minute bouts 40 (95% CI 17–63) (*p* = 0.001) (Table 3). Both counts and counts per minute of wear-time exhibited similar patterns to the steps and MVPA findings, with highly significant intervention effects at 3 months; at 12 months counts, but not counts per minute of wear-time, were statistically significant (Table 3).

**Table 3:** Treatment effect for primary and secondary outcome measures.

Outcome Measure	Control Group (Mean [SD])			Intervention Group (Mean [SD])			Treatment Effect at 3 Months^a^			Treatment Effect at 12 Months^a^		
	Baseline	3 Months	12 Months	Baseline	3 Months	12 Months	Effect	95% CI	*p*-Value	Effect	95% CI	*p*-Value
n	148	138	136	150	142	137	280			273		
Daily step count	7,380 (2,988)	6,904 (3,061)	6,872 (2,792)	7,314 (2,693)	7,903 (3,194)	7,514 (3,165)	1,037	(513–1,560)	<0.001	609	(104–1,115)	0.018
MVPA: Total weekly minutes	301 (169)	278 (169)	285 (174)	296 (154)	333 (185)	319 (188)	66	(36–96)	<0.001	40	(10–70)	0.009
MVPA: Total weekly minutes in ≥10 minute bouts	88 (113)	72 (102)	75 (108)	96 (104)	134 (138)	118 (130)	63	(40–87)	<0.001	40	(17–63)	0.001
Daily counts	246,610 (111,809)	231,278 (110,870)	239,158 (114,776)	244,225 (94,980)	266,357 (119,648)	257,511 (117,882)	40,459	(21,483–59,436)	<0.001	21,436	(2,207–40,665)	0.029
Counts per minute of wear-time	310 (129)	295 (127)	304 (136)	306 (112)	333 (140)	322 (139)	48	(25–70)	<0.001	23	(−0.7 to 46)	0.057

All accelerometry data are adjusted for day of the week and day order of wearing the accelerometer with participant as a random effect in a multi-level model.

^**a**^The treatment effect is the difference between groups (intervention − control) in change from baseline at 3 months and 12 months. The changes at 3 and 12 months are adjusted for baseline measure, practice, age, gender, month of baseline accelerometry, day of the week, and day order of wearing the accelerometer in a multi-level model with household and participant as random effects.

### Subgroup Analyses

**Fig 2:**
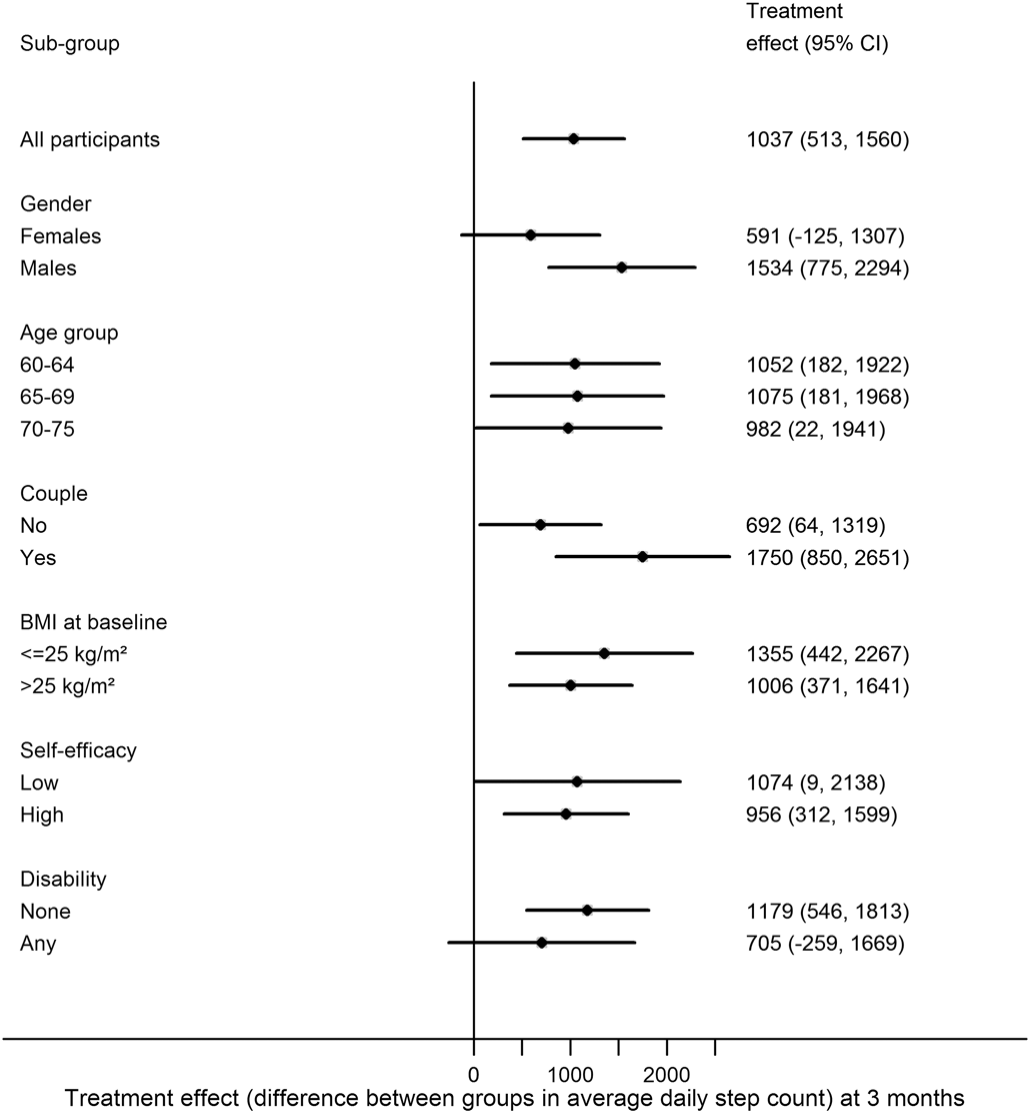
Treatment effect by subgroup at 3 months.

None of the potential effect modifiers examined (age, gender, taking part as a couple, BMI, exercise self-efficacy, disability) were statistically significant (Fig. 2). However, there was a suggestion that the treatment effect could be larger for men 1,534 (95% CI 775–2,294) than women 591 (95% CI −125 to 1,307) (*p* = 0.08) and stronger for couples 1,750 (95% CI 850–2,651) than individuals 692 (95% CI 64–1,319) (*p* = 0.06).

**Effect of the intervention on other health related outcomes.** There were no between-group differences in change in BMI, 0.001 kg/m^2^ (95% CI −0.17 to 0.18) (*p* = 0.98), or in change in fat mass, 0.39 kg (95% CI −0.85 to 0.07) (*p* = 0.10), at 3 months (Table 4). There were no between-group differences in mean scores of depression, anxiety, or pain at 3 or 12 months (Table 4).

**Table 4:** Other outcome measures.

Outcome Measure	Control Group (Mean [SD])			Intervention Group (Mean [SD])			Treatment Effect at 3 Months^a^			Treatment Effect at 12 Months^a^		
	Baseline	3 Months	12 Months	Baseline	3 Months	12 Months	Effect	95% CI	*p*-Value	Effect	95% CI	*p*-Value
n	148	143	137	150	146	136	240–288			239–267		
BMI (kg/m^2^)	27.1 (4.5)	26.8 (4.3)	—	27.7 (4.1)	27.6 (4.1)		0.00	(−0.17 to 0.18)	0.98	—		
Fat mass (kg)^**b**^	23.2 (9.7)	23.1 (9.7)	—	24.5 (8.5)	23.9 (8.6)	—	−0.39	(−0.85 to 0.07)	0.10	—		
Geriatric depression score (0–14)^**c**^	1.5 (1.9)	1.4 (2.3)	1.3 (2.0)	1.3 (2.0)	1.1 (1.8)	1.1 (1.7)	−0.24	(−0.58 to 0.09)	0.16	−0.23	(−0.59 to 0.14)	0.22
Fear anxiety score (0–3)^**c**^	0.6 (0.8)	0.6 (0.8)	0.7 (0.9)	0.6 (0.8)	0.6 (0.8)	0.6 (0.9)	−0.01	(−0.15 to 0.14)	0.93	−0.01	(−0.17 to 0.15)	0.94
Self−reported pain (0–3)^**c**^	0.9 (0.8)	1.0 (0.8)	0.9 (0.8)	1.0 (0.8)	1.0 (0.8)	0.9 (0.8)	0.01	(−0.15 to 0.18)	0.86	0.04	(−0.13 to 0.20)	0.69

^**a**^The treatment effect is the difference between groups (intervention-control) in change from baseline at 3 months and 12 months. The differences at 3 and 12 months are adjusted for baseline measure, practice, age, gender, and month of baseline accelerometry in a multi-level model with household as a random effect.

^**b**^Fat mass was missing for six and three participants in the control group and for five and seven participants in the intervention group at baseline and 3 months, respectively.

^**c**^Geriatric depression scores, fear anxiety scores, and self-reported pain were missing for varying numbers of participants (maximum 15) at each time-point.

### Effect of the Intervention on Adverse Events

Questionnaire systematically reported adverse events (both total number and individual types) were similar between the groups; intervention-control difference of total questionnaire adverse events −4% (95% CI −15 to 7) (*p* = 0.48) and −1.0% (95% CI −13 to 11) (*p* = 0.90) at 3 and 12 months, respectively (Table 5). Spontaneously reported events were less common compared with questionnaire reported events and they were non-significantly higher in the intervention group than the control group at 3 months (5% [95% CI −0.3% to 11] [*p* = 0.09]) and 12 months (7% [95% CI −0.6 to 14] [*p* = 0.09]) (Table 5). The total of any adverse events reported on the questionnaire or spontaneously was similar between the groups, intervention-control difference −2% (95% CI −13 to 9) (*p* = 0.78) and 1% (95% CI −11 to 13) (*p* = 0.90) at 3 and 12 months, respectively.

**Table 5:** Adverse events.

Adverse Event	0–3 Months							0–12 Months						
	Control (C)		Intervention (I)		Difference			Control (C)		Intervention (I)		Difference		
	*n*/*N*	(%)	*n*/*N*	%	I − C	95% CI	*p*-Value	*n*/*N*	%	*n*/*N*	%	I − C	95% CI	*p*-Value
Any adverse event reported on questionnaire	35/128	(27)	30/129	(23)	−4%	(−15 to 7)	0.48	70/127	(55)	66/122	(54)	−1%	(−13 to 11)	0.90
Falls inside the house	1/137	(0.7)	6/138	(4)	4%	(−0.4 to 8)	0.12	8/130	(6)	15/126	(12)	6%	(−1 to 13)	0.13
Falls outside the house	7/136	(5)	9/135	(7)	2%	(−4 to 8)	0.62	23/134	(17)	26/125	(21)	4%	(−6 to 13)	0.53
Fractures	2/130	(2)	4/131	(3)	2%	(−3 to 6)	0.68	4/125	(3)	5/117	(4)	1%	(−4 to 7)	0.74
Sprains or injuries	16/133	(12)	14/133	(11)	−2%	(−9 to 6)	0.85	30/125	(24)	32/123	(26)	2%	(−9 to 13)	0.77
Deterioration in health problems already present, since start of study	16/136	(12)	13/136	(10)	−2%	(−10 to 5)	0.70	32/130	(25)	34/127	(27)	2%	(−8 to 13)	0.78
Adverse event reported spontaneously	5/143	(3)	13/146	(9)	5%	(−0.3 to 11)	0.09	11/144	(8)	21/146	(14)	7%	(−0.6 to 14)	0.09
Any adverse event reported on questionnaire or spontaneously	36/128	(28)	34/130	(26)	−2%	(−13 to 9)	0.78	71/128	(55)	70/124	(56)	1%	(−11 to 13)	0.90

Denominators differ as not all questions were answered by all participants.

**Sensitivity analyses.** In analyses giving equal weight to each participant (irrespective of the number of days of PA data), the estimated treatment effect of 1,041 (95% CI 519–1,563) steps/day (*p <* 0.001) was almost identical to that in our primary analysis (S3 Table). Imputation assuming participants with missing data were missing at random also made little difference. Even assuming extreme differences in intervention and control participants with missing data did not alter the broad conclusions (S3 Table).

Analyses using a wear-time criteria of ≥600 minutes/day produced similar results to the ≥540 minute cut-off (S4 Table): at 3 months, 995 (95% CI 456–1,535) steps/day (*p <* 0.001), 66 (95% CI 42–89) minutes/week in ≥10 minute bouts (*p <* 0.001); and at 12 months, 599 (95% CI 70–1,128) steps/day (*p* = 0.026) and 40 (95% CI 16–64) minutes/week in ≥10 minute bouts (*p* = 0.001).

### Qualitative Evaluations

**Acceptability for trial participants.** Both improvers and non-improvers were very positive about most intervention components. This perspective included using the pedometers “*it was quite a revelation….quite fun actually”* (participant 18, female, improver, aged 63), accelerometer feedback on PA intensity *“the graph was particularly good*, *I was very interested to see that”* (participant 5, female, non-improver aged 75), and seeing the nurse *“the support of the nurse….it was very good….she brought positive things forward”* (participant 1, male, non-improver, aged 67). The handbook had a more mixed reception: some who had increased their walking found it valuable *“the handbook was very helpful*, *I did fill it in and it made me think about it and what I wanted to achieve”* (participant 15, female, aged 69); whilst others who had not increased walking were sometimes critical *“American managementese garbage”* (participant 2, male, aged 67). Those who had increased their walking made positive comments about the programme overall *“… the whole PACE-Lift thing … it’s had an effect on my life and I’m fitter for it”* (participant 26, male, aged 68) and *“how much I now enjoy walking*, *just for its own sake*, *which I wouldn’t have said a year ago”* (participant 27, male, aged 67).

**Acceptability for practice nurses.** The nurses were all enthusiastic about the intervention, including the monitors: *“the equipment was excellent*, *the pedometers*, *the accelerometers*, *excellent*, *excellent …”* (Nurse C); the feedback on PA intensity *“they love the diary*, *working out what they were doing at that time and the graph*, *didn’t they*?*”* (Nurse C); and the time they spent with participants *“to give them the quality of time that they have been given in this trial …”* (Nurse B). Of particular value to them was the BCT training *“that really helped me to listen and ask open questions … we’re using it*, *I’ve changed my nursing practice through it”* (Nurse A); *“to communicate with patients*, *actually figure out what their agenda is and help them achieve their goals”* (Nurse D).

**Barriers and facilitators to PA.** All participants talked about the different intervention elements (use of pedometer and accelerometers; the handbooks and diaries; the nurse consultation; the individualised goal setting and developing strategies for the future). The differences between our two groups were in the perception of these as enablers (or barriers) to increasing walking. Improvers were more positive about the use of the pedometer: *“well I think we were motivated because we had. . . little pace … step counters*, *you know on our side*, *and so of course we kept checking to make sure that you tried to achieve the. . . steps a day*, *and we sort of kept motivated”* (participant 13, female, aged 67); individualised goal setting and monitoring *“she was just very very positive*. *If we’d had a bad week or whatever*, *you know*, *it had gone down from the week before*, *she just said “Well look how well you’re doing*!*”* (participant 28, female, improver, aged 62); the appropriateness of walking as a form of PA for people of “their age” *“Walking certainly*, *because I mean … at my age*, *umm*, *virtually anything else is. . . inappropriate”* (participant 14, male, aged 71); and in developing strategies for keeping walking in the future “*Well it’s just … a habit we’ve got into I suppose and we just kept going from that*” (participant 29, male, aged 73). Both groups commented upon a range of constraints to increasing their walking with the weather “*Well*, *no*, *just the weather” (as a limitation on walking)* (participant 7, female, non-improver, aged 71) and existing health problems “*I mean I have high blood pressure and umm … uhh … osteoporosis so*, *in fact*, *you know*, *the walking benefits very much. . .”* (participant 22, female, improver, aged 65). However, as that participant illustrated, the improvers were able to overcome these barriers and saw the consequent benefits for other aspects of their life.

## Discussion

### Main Findings

In this trial, differences in both step-counts and time spent in MVPA (nearly all within bouts of ≥10 minutes) were observed between the intervention group compared with the control group at 3 months and at 12 months. No effect was seen on other health outcomes, including adverse events. The intervention was well accepted, with 87% attending all four nurse sessions, nearly all wearing the accelerometer before their appointments, and positive comments about all aspects of the trial from participants and nurses. While the intervention itself, particularly the use of accelerometer PA intensity feedback, was novel, the key methodological development was the successful embedding of the intervention within primary care. Primary care provided the sampling frame, an efficient mechanism for identifying unsuitable participants, practice nurses to deliver the intervention, and a supportive environment.

### Strengths and Weaknesses of This Study

To our knowledge, this study is the largest reported pedometer-based walking intervention for older people, recruiting from a population-based sample and with a high recruitment rate of 30%, higher than other primary care PA trials [22,23] and over 90% retention rate at 12 months. Detailed sensitivity analyses demonstrated the effect estimates were robust. Around half of participants were men, often under-represented in trials [8–10], and there was a balance across age groups. A study limitation is that whilst the intervention was effective in older men and women from an affluent, non-ethnically diverse socio-economic area, generalisability to more diverse populations cannot be assumed. Previously published work on those declining participation in this trial showed that participants were more likely than non-participants to live in affluent postcodes [24].

The fact that the intervention group at baseline was slightly older than the control group was consistent with their leaving full-time education younger and having slightly higher levels of chronic illness and disability. However, our analyses were based on change, with baseline level of PA being controlled for. Adjusting for baseline activity controls for many factors associated with activity in cross-sectional analyses. Moreover, our effect modification analyses suggests no difference in the intervention by age or disability.

In the study design, nurses from the practices, rather than researchers or exercise specialists, delivered the intervention and incorporated feedback on participants’ PA intensity from accelerometers to provide personalised PA goals. As the intervention involved multiple components, we cannot identify which components were important in changing PA, although qualitative evaluations from both participants and nurses emphasised the value of all the different intervention elements. Whilst researchers assessing outcomes were not masked to group status, an important study strength was that the main outcome was accelerometer PA measurement, enabling change in both step-counts and PA intensity to be objectively assessed. Waist-worn accelerometers measure walking accurately, while self-reported PA, particularly walking, is prone to recall bias [25]. One potential criticism in using monitors to assess PA is that participants might try harder when being monitored. However, any Hawthorne effect would have been reduced by our 7-day protocol and would have affected both groups [9]. The fact that the observed changes in the intervention group affected not only steps and MVPA but also bouts of MVPA of at least 10 minutes, suggests that participants were making recommended changes by increasing planned, structured walking.

A further strength is the inclusion of qualitative findings from intervention participants and practice nurses. The devices and nurse-delivered support were generally valued by all participants regardless of changes in their levels of activity. However, this raises the issue of why some participants engaged with the psychological techniques of goal setting and developing strategies to embed activity into their lives, whilst others did not. A potential weakness may be that we were unable to interview sufficient people, to reach saturation, and to gain a clearer picture of the factors supporting long-term behaviour change. Understanding why and how older people engage with increasing and maintaining changes in PA is likely to require a more complex mixed-methods design that examines individual psycho-social factors, the wider socio-environmental context, and other key determinants of health.

### Main Results in Context of Other Literature

Our difference between groups in average daily step-count of 1,037 (95% CI 513–1,560) is consistent with the PA increases reported in systematic reviews of pedometer-based interventions, although smaller than reviews reported (2,000–2,500 steps/day) [8–10]. This finding could reflect the fact that PACE-Lift was population-based rather than volunteer-based and that it measured step-counts using accelerometers, whereas the above studies used pedometers; there is no direct conversion factor [26]. A Japanese study of 68 older women showed that accelerometer feedback of PA intensity helped to increase PA levels [27]. Our study confirms the feasibility and effectiveness of accelerometer PA feedback and suggests this should be explored further.

Previous walking interventions demonstrating increased time spent in MVPA are based on self-report [28,29]. Recent trials using accelerometery have shown reductions in sedentary time [12] and increases in minutes spent walking [11], but have been based on very small numbers [11,12] or on women only [11]. There is evidence that people over-report PA, particularly walking [25], so our objective measure of an increase in weekly MVPA of 66 minutes, in a large trial including men and women, is an important finding. In terms of public health advice to achieve MVPA in bouts of at least 10 minutes [1], 63 of the 66 minutes weekly increase was in ≥10 minute bouts. Whilst subgroup differences were not statistically significant, our study was not powered to look directly at these interaction effects and our study suggests that the possibility of the intervention being more effective in men than women and in those attending as couples should not be ruled out. The gender effect is different from that suggested previously [8], where the effect size was greater for women; but authors urged caution owing to the lack of data on men. To our knowledge no other pedometer studies have compared effects in couples and individuals; household sampling enabled us to investigate this effect. We intended to sample, randomise, and provide the intervention by household cluster, but it turned out that most clusters were represented for randomisation and treatment by single individuals, which reduced our power for looking directly at the interaction effect of couple versus individual. We did not find a significant effect on anthropometric outcomes at 3 months, and we did not have funding for face-to-face assessments to measure weight and fat mass at 12 months. A meta-analysis of pedometer-based walking interventions and weight loss found that longer interventions and those with longer follow-up had greater effects, but the average increase in step-counts was 4,000 steps/day, which is much larger than in our study [30]. Our findings are consistent with another more recent pedometer-based walking intervention, which showed an effect on step-counts, but not on anthropometric measures [29]. No significant effect of the intervention on depression or anxiety scores either at 3 or 12 months was found. Whilst walking interventions have reported improved mood [31], published systematic reviews did not include this measure [8–10]. Other primary care pedometer-based walking interventions have not shown an effect on mental health [12], depression, or anxiety [11].

Whilst walking is safe [1,5], a large trial in 40–74 year old women, which promoted a single walking target, reported significantly more falls in the walking group [28]. Our trial did not find group differences in self-reported pain or adverse events. This finding may be due to our individualised PA plans based on the person’s baseline and “starting-low-and-going-slow” [5]. Mutrie and colleagues required participants to have a face-to-face check with a general practitioner (GP) at baseline, excluding 35% (167/461) and called for simpler, less time-consuming screening methods [12]. Our two-stage exclusion method, initially using computerised record Read codes and followed by GP examination of potential participant lists, excluding 15% and 9% of potential participants, respectively, was quick and easy to implement, did not require a GP visit, and raised no safety concerns. Our trial confirmed other findings that primary care nurses can be effective in increasing PA levels in older people [12,28,32] and provided this evidence using objective PA measures. Our nurse PA consultations followed primary care exercise prescribing guidance to chart progress, set goals, and solve problems [33]. This method fits with pedometer-based systematic review findings, suggesting that interventions with individual tailoring and personalised activity goals are more effective [10].

### Implications for Policy and Practice

The increase in PA levels in this trial was sufficiently large to have modest but appreciable effects on risks of important chronic diseases. Based on earlier reports quantifying the strengths of associations between PA (particularly walking) and coronary heart disease (CHD) [34] and Type 2 diabetes [35], the 40 minute weekly difference between the groups in walking observed at one year, if sustained, would be expected to reduce CHD risk by about 5.5% (95% CI 3.9%–6.7%) and type 2 diabetes risk by 9.1% (95% CI 4.5%–13.5%) (see S5 Text for full details of how risk reductions were estimated). Importantly, the reduction in risk of diabetes appears largely independent of any impact on BMI [35].

Our study demonstrates that practice nurses can safely deliver an intervention to increase objectively measured PA levels in older people at 3 months, with a sustained effect at 12 months. Moreover, this was an acceptable population-based intervention, with 30% uptake in the target age group. Researchers wishing to recruit older people to PA sudies should consider the two-stage primary care recruitment method described here, which provides a population-based sample, estimation of recruitment rate, and generalisability, with an efficient, safe way of excluding unsuitable patients. However, the main advantage that primary care offers is an ideal setting for delivering PA interventions in this age group and the opportunity to integrate this into routine care. Our intervention required four PA consultations, each longer than standard practice nurse consultations and training for practice nurses both in using accelerometers and in BCTs. This method has implications for primary care, in that effective delivery of interventions that produce sustained objective PA increases, involving personalised activity goals and feedback of PA intensity, requires more time, training, and support than is currently standard in NHS Health Checks [7] or routine care.

### Unanswered Questions and Future Research

Future trials need to distinguish the different aspects of PA interventions, to understand the effects of the pedometer or accelerometer separately from BCTs and support offered [9,10]. They also need to test interventions in socio-economically diverse populations, and with older people who have higher levels of morbidity and disability. Inclusion of a qualitative dimension to future trials would provide further valuable insights into how older people can embrace PA as part of their daily lives. Robust evidence on costs and cost-effectiveness needs to be gathered before this or similar programmes can be implemented on a larger scale in routine health care. Following demonstration of the effectiveness of this intervention, funding was obtained for a post hoc evaluation of costs and cost-effectiveness, using existing trial evidence, and is currently in process. Increased PA at 3 months is a significant finding, but maintenance of change is of key importance. Our findings that over 50% of the difference at 3 months was maintained at 12 months, 9 months post-intervention, is an important addition to the literature. Longer term follow-up to evaluate the need for maintenance support is needed.

## Supporting Information

S1 Fig Residuals from 3 month models for steps and weekly MVPA in ≥10 minute bouts. (a) Steps model: Distribution of residuals from steps model. (b) Steps model: Standardised normal probability plot of residuals. (c) Weekly MVPA in ≥10 minute bouts: Distribution of residuals. (d) Weekly MVPA in ≥10 minute bouts: Standardised normal probability plot of residuals.
(TIF)

S2 Fig Residuals from 12 month models for steps and weekly MVPA in ≥10 minute bouts. (a) Steps model: Distribution of residuals from steps model. (b) Steps model: Standardised normal probability plot of residuals. (c) Weekly MVPA in ≥10 minute bouts: Distribution of residuals. (d) Weekly MVPA in ≥10 minute bouts: Standardised normal probability plot of residuals.
(TIF)

S1 Table Number of days with ≥540 minutes accelerometer recording time by treatment group at each assessment. (DOCX)

S2 Table Multi-level model for primary outcome (step-count at 3 months) with treatment effect and parameter estimates. (DOCX)

S3 Table Imputation and sensitivity analyses for primary outcome (step-counts at 3 months and 12 months). (DOCX)

S4 Table Treatment effect for primary and secondary outcome measures (600 minutes wear-time). (DOCX)

S1 Text PACE-Lift trial protocol. (PDF)

S2 Text PACE-Lift CONSORT checklist. (DOCX)

S3 Text PACE-Lift patient handbook. (PDF)

S4 Text PACE-Lift physical activity diary. (PDF)

S5 Text Estimating reduction in risk of coronary heart disease and type 2 diabetes. (DOCX)

## Abbreviations

BCT: behaviour change technique
BMI: body mass index
MVPA: moderate to vigorous intensity physical activity
NHS: National Health Service
PA: physical activity

## References

[1] Department of Health (2011) Start Active, Stay Active: A report on physical activity for health from the four home countries' Chief Medical Officers. London: UK Department of Health.

[2] Garber CE, Blissmer B, Deschenes MR, Franklin BA, Lamonte MJ, et al (2011) American College of Sports Medicine position stand. Quantity and quality of exercise for developing and maintaining cardiorespiratory, musculoskeletal, and neuromotor fitness in apparently healthy adults: guidance for prescribing exercise. Med Sci Sports Exerc 43: 1334–1359. 10.1249/MSS.0b013e318213fefb 21694556

[3] Joint Health Surveys Unit (2009) Health Survey for England 2008 Physical activity & fitness. London: The NHS Information Centre for Health and Social Care 10.1186/1756-0500-7-921

[4] Harris TJ, Owen CG, Victor CR, Adams R, Cook DG (2009) What factors are associated with physical activity in older people, assessed objectively by accelerometry? Br J Sports Med 43: 442–450. 10.1136/bjsm.2008.048033 18487253

[5] Cress ME, Buchner DM, Prohaska T, Rimmer J, Brown M et al (2005) Best practices for physical activity programs and behavior counseling in older adult populations. J Aging Phys Act 13: 61–74. 15677836 10.1123/japa.13.1.61

[6] Ogilvie D, Foster CE, Rothnie H, Cavill N, Hamilton V, et al (2007) Interventions to promote walking: systematic review. BMJ 334: 1204 17540909 10.1136/bmj.39198.722720.BE PMC1889976

[7] NHS Health Checks Programme (2009) Putting prevention first: NHS Health Checks: Vascular Risk Assessment and Management Best Practice Guidelines. London: UK Department of Health.

[8] Kang M, Marshall SJ, Barreira TV, Lee JO (2009) Effect of pedometer-based physical activity interventions: a meta-analysis. Res Q Exerc Sport 80: 648–655. 19791652 10.1080/02701367.2009.10599604

[9] Bravata DM, Smith-Spangler C, Sundaram V, Gienger AL, Lin N et al (2007) Using pedometers to increase physical activity and improve health: a systematic review. JAMA 298: 2296–2304. 18029834 10.1001/jama.298.19.2296

[10] Hobbs N, Godfrey A, Lara J, Errington L, Meyer TD, et al (2013) Are behavioral interventions effective in increasing physical activity at 12 to 36 months in adults aged 55 to 70 years? A systematic review and meta-analysis. BMC Med 11: 75. 1741–7015–11–75 [pii];10.1186/1741–7015–11–75 [doi]. 10.1186/1741-7015-11-75 23506544 PMC3681560

[11] McMurdo ME, Sugden J, Argo I, Boyle P, Johnston DW, et al (2010) Do pedometers increase physical activity in sedentary older women? A randomized controlled trial. J Am Geriatr Soc 58: 2099–2106. 10.1111/j.1532-5415.2010.03127.x 21054290

[12] Mutrie N, Doolin O, Fitzsimons CF, Grant PM, Granat M, et al (2012) Increasing older adults' walking through primary care: results of a pilot randomized controlled trial. Fam Pract 29: 633–642. 10.1093/fampra/cms038 22843637 PMC3501246

[13] Harris T, Kerry S, Victor CR, Ekelund U, Woodcock A, et al (2013) Randomised controlled trial of a complex intervention by primary care nurses to increase walking in patients aged 60–74 years: protocol of the PACE-Lift (Pedometer Accelerometer Consultation Evaluation—Lift) trial. BMC Public Health 13: 5 10.1186/1471-2458-13-5 23289648 PMC3543841

[14] Freedson PS, Melanson E, Sirard J (1998) Calibration of the Computer Science and Applications, Inc. accelerometer. Med Sci Sports Exerc 30: 777–781. 9588623 10.1097/00005768-199805000-00021

[15] Trost SG, McIver KL, Pate RR (2005) Conducting accelerometer-based activity assessments in field-based research. Med Sci Sports Exerc 37: S531–S543. 16294116 10.1249/01.mss.0000185657.86065.98

[16] Katzmarzyk PT, Champagne CM, Tudor-Locke C, Broyles ST, Harsha D, et al (2011) A short-term physical activity randomized trial in the Lower Mississippi Delta. PLoS ONE 6: e26667 10.1371/journal.pone.0026667 22046325 PMC3201968

[17] Miller GD, Jakicic JM, Rejeski WJ, Whit-Glover MC, Lang W, et al (2012) Effect of varying accelerometry criteria on physical activity: The Look AHEAD Study. Obesity (Silver Spring) 21: 32–44. 10.1038/oby.2012.118 PMC3430806 23505166

[18] Boutron I, Moher D, Altman DG, Schulz KF, Ravaud P (2008) Extending the CONSORT statement to randomized trials of nonpharmacologic treatment: explanation and elaboration. Ann Intern Med 148: 295–309. 18283207 10.7326/0003-4819-148-4-200802190-00008

[19] Vickers AJ, Altman DG (2001) Statistics notes: analysing controlled trials with baseline and follow up measurements. BMJ 323: 1123–1124. 11701584 10.1136/bmj.323.7321.1123 PMC1121605

[20] Egbewale BE, Lewis M, Sim J (2014) Bias, precision and statistical power of analysis of covariance in the analysis of randomized trials with baseline imbalance: a simulation study. BMC Med Res Methodol 14: 49 10.1186/1471-2288-14-49 24712304 PMC3986434

[21] Noble M, McLennan D, Wilkinson K, Whitworth A, Barnes H (2008) The English Indices of Deprivation 2007. London: UK Department of Communities and Local Government, London.

[22] Tully MA, Cupples ME, Chan WS, McGlade K, Young IS (2005) Brisk walking, fitness, and cardiovascular risk: a randomized controlled trial in primary care. Prev Med 41: 622–628. 15917061 10.1016/j.ypmed.2004.11.030

[23] Sugden JA, Sniehotta FF, Donnan PT, Boyle P, Johnston DW, et al (2008) The feasibility of using pedometers and brief advice to increase activity in sedentary older women—a pilot study. BMC Health Serv Res 8: 169 10.1186/1472-6963-8-169 18691392 PMC2527003

[24] Rogers A, Harris T, Victor C, Woodcock A, Limb E, et al (2014) Which older people decline participation in a primary care trial of physical activity and why: insights from a mixed methods approach. BMC Geriatr 14: 46 10.1186/1471-2318-14-46 24725730 PMC3991893

[25] Tudor-Locke CE, Myers AM (2001) Challenges and opportunities for measuring physical activity in sedentary adults. Sports Med 31: 91–100. 11227981 10.2165/00007256-200131020-00002

[26] Tudor-Locke C, Craig CL, Aoyagi Y, Bell RC, Croteau KA, et al (2011) How many steps/day are enough? For older adults and special populations. Int J Behav Nutr Phys Act 8: 80 10.1186/1479-5868-8-80 21798044 PMC3169444

[27] Koizumi D, Rogers NL, Rogers ME, Islam MM, Kusunoki M, et al (2009) Efficacy of an accelerometer-guided physical activity intervention in community-dwelling older women. J Phys Act Health 6: 467–474. 19842461 10.1123/jpah.6.4.467

[28] Lawton BA, Rose SB, Elley CR, Dowell AC, Fenton A, et al (2008) Exercise on prescription for women aged 40–74 recruited through primary care: two year randomised controlled trial. BMJ 337: a2509 10.1136/bmj.a2509 19074218 PMC2769033

[29] Fitzsimons CF, Baker G, Gray SR, Nimmo MA, Mutrie N (2012) Does physical activity counselling enhance the effects of a pedometer-based intervention over the long-term: 12-month findings from the Walking for Wellbeing in the west study. BMC Public Health 12: 206 10.1186/1471-2458-12-206 22429600 PMC3349531

[30] Richardson CR, Newton TL, Abraham JJ, Sen A, Jimbo M, et al (2008) A meta-analysis of pedometer-based walking interventions and weight loss. Ann Fam Med 6: 69–77. 10.1370/afm.761 18195317 PMC2203404

[31] Murphy M, Nevill A, Neville C, Biddle S, Hardman A (2002) Accumulating brisk walking for fitness, cardiovascular risk, and psychological health. Med Sci Sports Exerc 34: 1468–1474. 12218740 10.1097/00005768-200209000-00011

[32] Stevens Z, Barlow C, Kendrick D, Masud T, Skelton DA, et al (2013) Effectiveness of general practice-based physical activity promotion for older adults: systematic review. Prim Health Care Res Dev 1–12. 10.1017/S1463423613000017 23506656

[33] Khan KM, Weiler R, Blair SN (2011) Prescribing exercise in primary care. BMJ 343: d4141 10.1136/bmj.d4141 21765112

[34] Zheng H, Orsini N, Amin J, Wolk A, Nguyen VT, et al (2009) Quantifying the dose-response of walking in reducing coronary heart disease risk: meta-analysis. Eur J Epidemiol 24: 181–192. 10.1007/s10654-009-9328-9 19306107

[35] Jeon CY, Lokken RP, Hu FB, van Dam RM (2007) Physical activity of moderate intensity and risk of type 2 diabetes: a systematic review. Diabetes Care 30: 744–752. 17327354 10.2337/dc06-1842

