# Supplementary figures and images for: A Primary Care Nurse-Delivered Walking Intervention in Older Adults: PACE (Pedometer Accelerometer Consultation Evaluation)-Lift Cluster Randomised Controlled Trial
Source: PLoS Med. 2015 Feb 17;12(2):e1001783. doi: 10.1371/journal.pmed.1001783 (PMC4331517; doi:10.1371/journal.pmed.1001783)

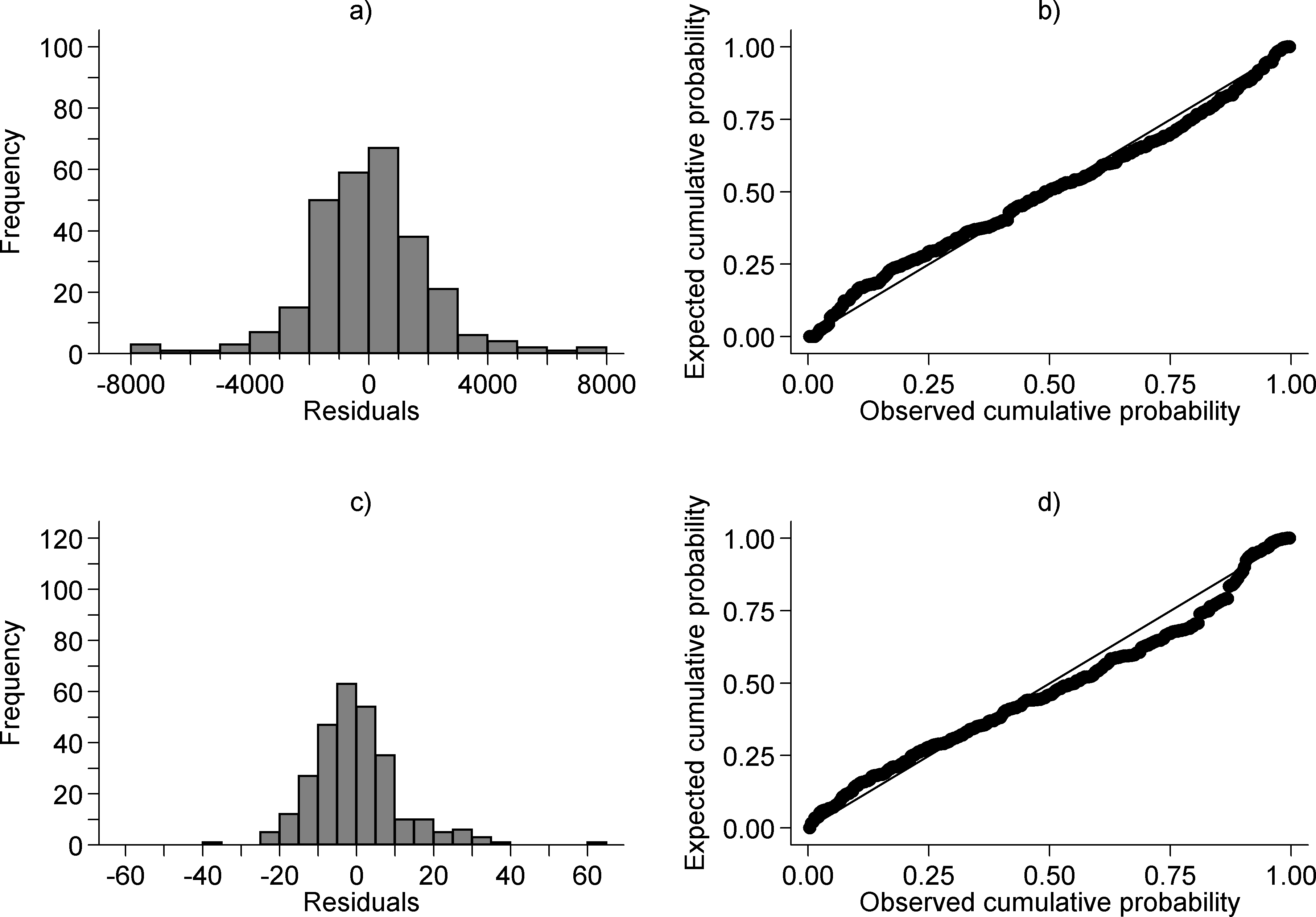

Supplement: S1 Fig — (a) Steps model: Distribution of residuals from steps model. (b) Steps model: Standardised normal probability plot of residuals. (c) Weekly MVPA in ≥10 minute bouts: Distribution of residuals. (d) Weekly MVPA in ≥10 minute bouts: Standardised normal probability plot of residuals. (TIF) [file pmed.1001783.s001.tif]

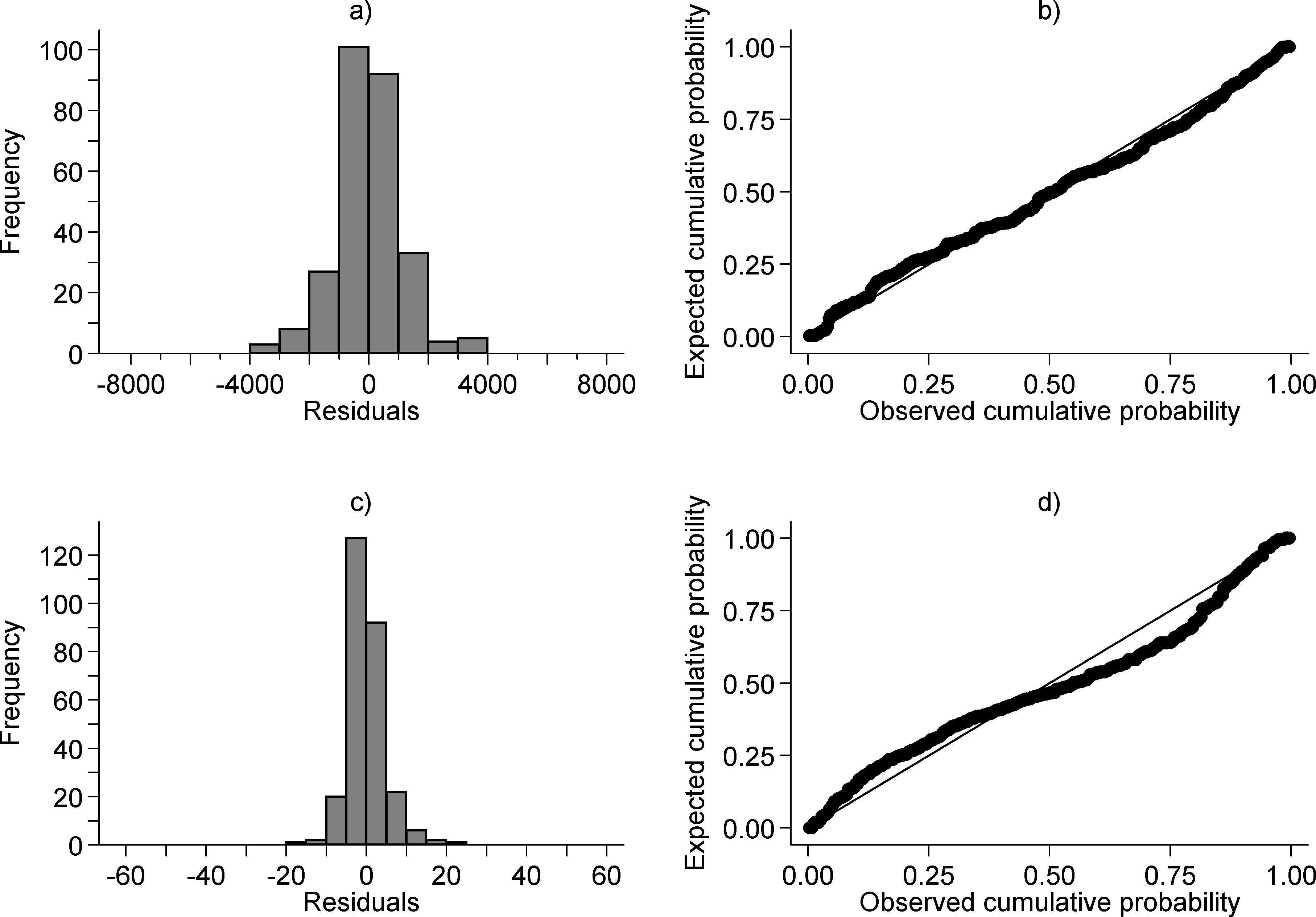

Supplement: S2 Fig — (a) Steps model: Distribution of residuals from steps model. (b) Steps model: Standardised normal probability plot of residuals. (c) Weekly MVPA in ≥10 minute bouts: Distribution of residuals. (d) Weekly MVPA in ≥10 minute bouts: Standardised normal probability plot of residuals. (TIF) [file pmed.1001783.s002.tif]
