# Supplementary material for: A Primary Care Nurse-Delivered Walking Intervention in Older Adults: PACE (Pedometer Accelerometer Consultation Evaluation)-Lift Cluster Randomised Controlled Trial
Source: PLoS Med. 2015 Feb 17;12(2):e1001783. doi: 10.1371/journal.pmed.1001783 (PMC4331517; doi:10.1371/journal.pmed.1001783)
Supplement: S1 Table — (DOCX) [file pmed.1001783.s003.docx]

**Table S1. Numbers of days with ≥540 minutes accelerometer recording time by treatment group at each assessment**

|  | **Baseline** | | | | **3 months** | | | | **12 months** | | | |
| --- | --- | --- | --- | --- | --- | --- | --- | --- | --- | --- | --- | --- |
|  | **Control (N=148)** | | **Intervention (N=150)** | | **Control (N=138)** | | **Intervention (N=142)** | | **Control (N=136)** | | **Intervention (N=137)** | |
|  | **n** | **(%)** | **n** | **(%)** | **n** | **(%)** | **n** | **(%)** | **n** | **(%)** | **n** | **(%)** |
| 1 day | 0 |  | 0 |  | 1 | (1) | 0 |  | 0 |  | 0 |  |
| 2 days | 0 |  | 0 |  | 2 | (1) | 2 | (1) | 1 | (1) | 1 | (1) |
| 3 days | 0 |  | 0 |  | 3 | (2) | 1 | (1) | 3 | (2) | 2 | (1) |
| 4 days | 0 |  | 0 |  | 5 | ( 4) | 5 | (4) | 7 | (5) | 4 | (3) |
| 5 days | 10 | (7) | 10 | (7) | 15 | (11) | 5 | (4) | 12 | (9) | 11 | (8) |
| 6 days | 27 | (18) | 24 | (16) | 25 | (18) | 32 | (23) | 31 | (23) | 29 | (21) |
| 7 days | 111 | (75) | 116 | (77) | 87 | (63) | 97 | (68) | 82 | (60) | 90 | (66) |
|  |  |  |  |  |  |  |  |  |  |  |  |  |
| **≥3 days** | **148** | **(100)** | **150** | **(100)** | **135** | **(98)** | **140** | **(99)** | **135** | **(99)** | **136** | **(99)** |
| **≥5 days** | **148** | **(100)** | **150** | **(100)** | **127** | **(92)** | **134** | **(94)** | **125** | **(92)** | **130** | **(95)** |
