# Supplementary material for: A Primary Care Nurse-Delivered Walking Intervention in Older Adults: PACE (Pedometer Accelerometer Consultation Evaluation)-Lift Cluster Randomised Controlled Trial
Source: PLoS Med. 2015 Feb 17;12(2):e1001783. doi: 10.1371/journal.pmed.1001783 (PMC4331517; doi:10.1371/journal.pmed.1001783)
Supplement: S2 Table — (DOCX) [file pmed.1001783.s004.docx]

**Table S2. Multi-level model for primary outcome (step-count at 3 months) with treatment effect and parameter estimates**

| **Fixed effect parameters** | |  |  |  |
| --- | --- | --- | --- | --- |
| **Dependent variable** | | **β coefficient** | **95% confidence interval** | **p-value** |
|  | |  |  |  |
| **Household level fixed effects** | |  |  |  |
| **Treatment group** | |  |  |  |
|  | Control | --- |  |  |
|  | Intervention | 1037 | (513, 1560) | <0.001 |
| **General practice** | |  |  |  |
|  | 1 | --- |  |  |
|  | 2 | -1010 | (-1649, -371) | 0.002 |
|  | 3 | -7 | (-665, 651) | 0.983 |
|  | |  |  |  |
| **Individual level fixed effects** | |  |  |  |
| **Adjusted baseline steps** | | 0.75 | (0.66, 0.85) | <0.001 |
| **Gender** | |  |  |  |
|  | Female | --- |  |  |
|  | Male | 106 | (-407, 620) | 0.685 |
| **Age at randomisation** | |  |  |  |
|  | 60-64 years | --- |  |  |
|  | 65-69 years | -140 | (-773, 493) | 0.665 |
|  | 70-75 years | -613 | (-1282, 56) | 0.072 |
| **Month of baseline accelerometry** | |  |  |  |
|  | January | --- |  |  |
|  | February | -1492 | (-2940, -45) | 0.043 |
|  | March | -2044 | (-3492, -597) | 0.006 |
|  | April | -2457 | (-4005, -909) | 0.002 |
|  | May | -1917 | (-3515, -320) | 0.019 |
|  | June | -1708 | (-3072, -345) | 0.014 |
|  | July | -1968 | (-3535, -402) | 0.014 |
|  | August | -2834 | (-4293, -1376) | <0.001 |
|  | September | -3736 | (-5391, -2080) | <0.001 |
|  | October | -2139 | (-3550, -727) | 0.003 |
|  | November | -1580 | (-2978, -181) | 0.027 |
|  | December | -248 | (-1796, 1300) | 0.754 |
|  |  |  |  |  |
| **Day level fixed effects** | |  |  |  |
| **3 month accelerometry: day of the week** | |  |  |  |
|  | Monday | --- |  |  |
|  | Tuesday | 15 | (-504, 534) | 0.954 |
|  | Wednesday | -58 | (-570, 453) | 0.824 |
|  | Thursday | 323 | (-198, 845) | 0.224 |
|  | Friday | 10 | (-510, 529) | 0.971 |
|  | Saturday | -25 | (-539, 490) | 0.926 |
|  | Sunday | -797 | (-1320, -273) | 0.003 |
| **3 month accelerometry: day of wear** | |  |  |  |
|  | 1 | -- |  |  |
|  | 2 | 385 | (-139, 910) | 0.150 |
|  | 3 | -102 | (-619, 415) | 0.699 |
|  | 4 | -305 | (-835, 224) | 0.259 |
|  | 5 | -129 | (-656, 398) | 0.631 |
|  | 6 | -340 | (-862, 182) | 0.202 |
|  | 7 | -119 | (-652, 414) | 0.661 |
|  |  |  |  |  |
| **Constant** | | 3835 | (2236, 5434) | <0.001 |
|  | |  |  |  |
| **Random effect parameters** | | **SD** | **95% confidence interval** |  |
| Household | | 219 | (0.0002, 2.3 x 10^8^) |  |
| Individual | | 1793 | (1407, 2284) |  |
|  | |  |  |  |
| Residual | | 2884 | (2783, 2989) |  |
