# Supplementary material for: A Primary Care Nurse-Delivered Walking Intervention in Older Adults: PACE (Pedometer Accelerometer Consultation Evaluation)-Lift Cluster Randomised Controlled Trial
Source: PLoS Med. 2015 Feb 17;12(2):e1001783. doi: 10.1371/journal.pmed.1001783 (PMC4331517; doi:10.1371/journal.pmed.1001783)
Supplement: S3 Table — (DOCX) [file pmed.1001783.s005.docx]

**Table S3. Imputation and sensitivity analyses for primary outcome (step counts at 3 months and 12 months)**

|  |  | **Treatment effect at 3 months** | | | **Treatment effect at 12 months** | | |
| --- | --- | --- | --- | --- | --- | --- | --- |
| **Analysis based on all participants with follow-up data** | | **Effect** | **95% CI** | ***p*-value** | **Effect** | **95% CI** | ***p*-value** |
|  | Model using daily step count, up to 7 observations per participant | 1037 | (513, 1560) | <0.001 | 609 | (104, 1115) | 0.018 |
|  | Model using adjusted average daily step count, 1 observation per participant | 1041 | (519, 1563) | <0.001 | 610 | (104, 1117) | 0.018 |
|  |  |  |  |  |  |  |  |
| **Missing at random.** | |  |  |  |  |  |  |
| Analysis conditional on covariates. Models using imputed step counts for participants with no follow-up data. | |  |  |  |  |  |  |
|  | Imputed using treatment group, baseline steps, gender, age, practice, month of baseline accelerometry | 1051 | (532, 1571) | <0.001 | 607 | (90, 1124) | 0.021 |
|  | Imputed using treatment group, baseline steps, gender, age, practice, month of baseline accelerometry, IMD and self-reported pain | 1050 | (521, 1579) | <0.001 | 605 | (97, 1112) | 0.020 |
|  | Imputed using treatment group, baseline steps, gender, age, practice, month of baseline accelerometry, IMD, self-reported pain and fat mass | 1049 | (531, 1567) | <0.001 | 599 | (95, 1102) | 0.020 |
|  |  |  |  |  |  |  |  |
| **Missing not at random.** | |  |  |  |  |  |  |
| Analysis based on extreme assumptions for missing data. Change in mean steps in control group from baseline to 3 and 12 months is 0. | |  |  |  |  |  |  |
| Change in mean steps in treatment group from baseline to 3 months | |  |  |  |  |  |  |
|  | -3000 | 848 | (326, 1370) | 0.001 |  |  |  |
|  | 0 | 1008 | (486, 1530) | <0.001 |  |  |  |
|  | +3000 | 1168 | (646, 1690) | <0.001 |  |  |  |
|  |  |  |  |  |  |  |  |
| Change in mean steps in treatment group from baseline to 12 months | |  |  |  |  |  |  |
|  | -1500 |  |  |  | 585 | (79, 1092) | 0.023 |
|  | 0 |  |  |  | 715 | (209, 1222) | 0.006 |
|  | +1500 |  |  |  | 845 | (339, 1352) | 0.001 |

The treatment effect is the difference between groups (Intervention – Control) in the change from baseline at 3 months and 12 months.

Imputations were carried out using *mi impute* in Stata, with 20 iterations and defining the random seed. xtmixed models were then fitted using *mi estimate: xtmixed …..*
