# Supplementary material for: A Primary Care Nurse-Delivered Walking Intervention in Older Adults: PACE (Pedometer Accelerometer Consultation Evaluation)-Lift Cluster Randomised Controlled Trial
Source: PLoS Med. 2015 Feb 17;12(2):e1001783. doi: 10.1371/journal.pmed.1001783 (PMC4331517; doi:10.1371/journal.pmed.1001783)
Supplement: S4 Table — (DOCX) [file pmed.1001783.s006.docx]

**Table S4. Treatment effect for primary and secondary outcome measures (600 minutes wear time)**

|  | **Control Group (Mean (sd))** | | | **Intervention Group (Mean (sd))** | | | **Treatment effect at 3 months*** | | | **Treatment effect at 12 months*** | | |
| --- | --- | --- | --- | --- | --- | --- | --- | --- | --- | --- | --- | --- |
|  | **Baseline** | **3 months** | **12 months** | **Baseline** | **3 months** | **12 months** | **Effect** | **95% CI** | ***p*-value** | **Effect** | **95% CI** | ***p*-value** |
| **N** | 143 | 135 | 133 | 143 | 136 | 131 | 271 |  |  | 273 |  |  |
|  | | | | | | | | | | | | |
| **Daily step count** | | | | | | | | | | | | |
|  | 7498 | 7063 | 6975 | 7474 | 8036 | 7693 | 995 | (456, 1535) | <0.001 | 599 | (70, 1128) | 0.026 |
|  | (3000) | (3129) | (2784) | (2654) | (3163) | (3148) |  |  |  |  |  |  |
|  | | | | | | | | | | | | |
| **Moderate or Vigorous Physical Activity: Total weekly minutes** | | | | | | | | | | | | |
|  | 308 | 287 | 292 | 302 | 339 | 327 | 64 | (33, 95) | <0.001 | 39 | (7, 71) | 0.016 |
|  | (171) | (172) | (175) | (155) | (185) | (189) |  |  |  |  |  |  |
|  | | | | | | | | | | | | |
| **Moderate or Vigorous Physical Activity: Total weekly minutes in ≥ 10 minute bouts** | | | | | | | | | | | | |
|  | 91 | 73 | 77 | 98 | 137 | 121 | 66 | (42, 89) | <0.001 | 40 | (16, 64) | 0.001 |
|  | (115) | (103) | (109) | (104) | (137) | (131) |  |  |  |  |  |  |
|  | | | | | | | | | | | | |
| **Daily counts** | | | | | | | | | | | | |
|  | 251,904 | 237,190 | 243,884 | 248,229 | 270,563 | 262,405 | 40,806 | (21,482, 60,130) | <0.001 | 21,132 | (861, 41,402) | 0.041 |
|  | (111,918) | (111,655) | (114,155) | (94,833) | (119,821) | (118,108) |  |  |  |  |  |  |
|  | | | | | | | | | | | | |
| **Counts per minute of wear time** | | | | | | | | | | | | |
|  | 314 | 299 | 306 | 307 | 333 | 324 | 45 | (22, 69) | <0.001 | 22 | (-3, 47) | 0.080 |
|  | (130) | (128) | (136) | (112) | (139) | (138) |  |  |  |  |  |  |

*All accelerometry data are adjusted for day of the week and day order of wearing the accelerometer with participant as a random effect in a multi-level model.*

** The treatment effect is the difference between groups (intervention – control) in change from baseline at 3 months and 12 months. The changes at 3 and 12 months are adjusted for baseline measure, practice, age, gender, month of baseline accelerometry, day of the week and day order of wearing the accelerometer in a multi-level model with household and participant as random effects.*
