# Supplementary material for: A Primary Care Nurse-Delivered Walking Intervention in Older Adults: PACE (Pedometer Accelerometer Consultation Evaluation)-Lift Cluster Randomised Controlled Trial
Source: PLoS Med. 2015 Feb 17;12(2):e1001783. doi: 10.1371/journal.pmed.1001783 (PMC4331517; doi:10.1371/journal.pmed.1001783)
Supplement: S3 Text — (PDF) [file pmed.1001783.s009.pdf]

Improving Health: Changing Behaviour

PACE-Lift Patient Handbook

PACE-lift

Contents

	Page
1. Section 1: Session one - First steps	3
2. Section 2: Session two - Continuing the changes	15
3. Section 3: Session three – Keeping up the changes	23
4. Section 4: Session four – Building lasting habits	33
5. Section 5: Walking planners	42
6. Section 6: Physical activity & step-count diary sheets	46

Acknowledgements:

This PACE-Lift patient handbook has been adapted from the NHS Health Trainer Handbook (2008) written by the British Psychological Society, Division of Health Psychology Team (Susan Michie, Nichola Rumsey, Anna Fussell, Wendy Hardeman, Marie Johnston, Stanton Newman, Lucy Yardley).
It has been adapted for the PACE-Lift trial of physical activity in older people by Lee David, Debbie Brewin, Alison Woodcock and Tess Harris (contact tharris@sgul.ac.uk) funded by the Research for Patient Benefit Programme. It was printed by Media Services at St George's University, of London (2011).

Session One: First steps

Background information on your health

A. Health conditions I have that could benefit from me doing more physical activity

(E.g. high blood pressure, diabetes, heart disease, stroke, arthritis, depression, asthma, chronic bronchitis, being overweight)

____

____

____

B. Problems I have that could make doing more physical activity difficult for me

(E.g. back pain, foot pain, breathlessness, chest pain, palpitations, weakness, dizziness)
Some of these answers may be the same as in A

____

____

____

Note: If doing more walking or other physical activities gives you chest pain, palpitations, dizziness or makes you feel faint or fall over, you should stop doing them and tell your GP

The benefits of increasing your physical activity

Reduce the impact of health problems

Physical activity can prevent or improve:

Heart disease, Stroke

Diabetes, High blood pressure

Osteoporosis, Several cancers

Arthritis and back pain

Improve wellbeing

Relieve stress, anxiety and depression

Make you feel happier

Increase your energy and fitness

Improve sleep

Get you out of the house meeting people

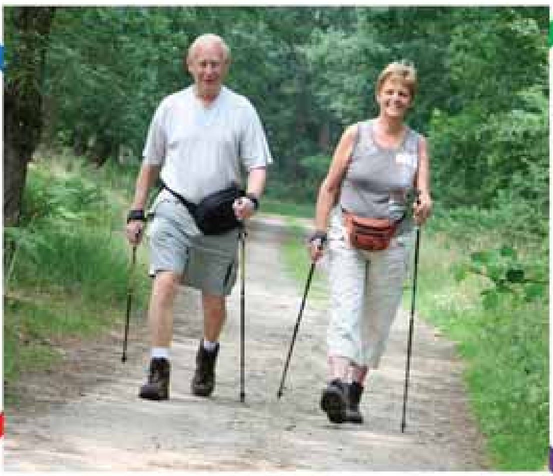

Reduce some of the effects of ageing

Improve flexibility and balance

Protect against developing dementia

Maintain mobility and being able to live independently

Help maintain a healthy weight

Physical activity can:

Help to maintain a healthy weight

Prevent obesity

Which of these benefits are most important or meaningful for you?

Summary of your current physical activity levels

A. Information from the questionnaires

Walking outside approximately____________________________ minutes per week

Cycling approximately ____________________________________ minutes per week

Swimming approximately _________________________________ minutes per week

Gardening approximately _________________________________ minutes per week

Other physical activity ________________ approximately _____ minutes per week

B. Information from the accelerometer

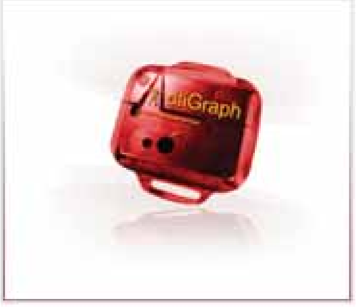

The accelerometer measures the **number of steps** that you take and also the **intensity** of your physical activity:

Sedentary activity e.g. sitting, watching television, lying down

Light activity e.g. walking slowly, strolling, light housework

Moderate activity - makes you feel a little warm and breathe a bit harder than normal, but you should still be able to talk whilst doing the activity (E.g. walking briskly, cycling or swimming at a regular pace, heavy housework eg hoovering)

Vigorous or very vigorous activity - makes you sweaty and makes you breathe very hard, you will be unable to talk whilst doing it. (E.g. jogging, aerobics, digging, fast cycling or swimming)

The accelerometer showed that over 7 days you spent the following time in these activities:

Sedentary activity _________ minutes   Light activity _____________ minutes

Moderate activity _________ minutes   Vigorous activity __________ minutes

At least moderate activity (moderate + vigorous) _________ minutes

Average number of daily steps ____________________________________

How much physical activity is good for older people?

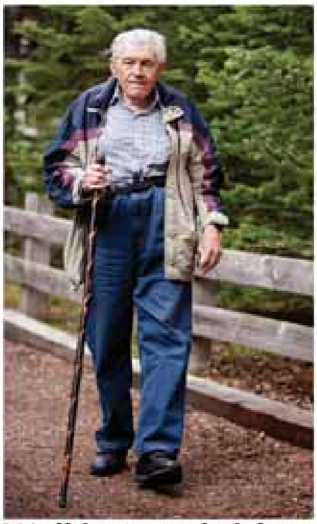

For health benefits, adults and older adults should do at least 30 minutes per day on at least 5 days per week (i.e. 150 minutes per week) of at least moderate intensity activity.

The 30 minutes does not need to be done all at once, it can be broken down, but it should be in bouts of at least 10 minutes each.

Some people may not be able to achieve these levels because of disabilities or problems with their health, or lack of fitness.

Walking at a brisk pace (approximately 3miles or 5km per hour) counts as moderate intensity activity; slower walking counts as light activity. Walking is an ideal activity for older people, as it has a very low risk of harm and can be built up gradually.

How can you increase your walking in a safe way?

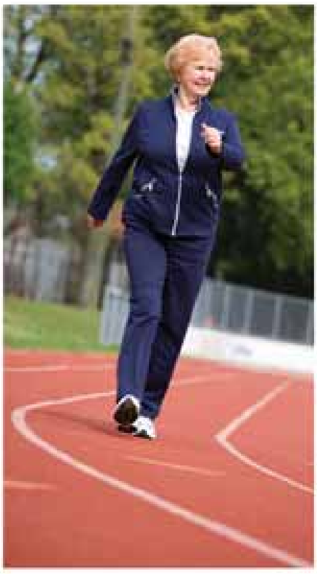

- Start low-and-go-slow! This means that you should start from where **you** are at the moment and make changes in a gradual way.
- Start by increasing the amount of activities that you already do, like walking, rather than taking up new activities.
- Gradually increase both the amount of walking that you do (the number of steps per day) and the intensity (how fast you walk).
- Wear sturdy shoes or trainers when you walk. You will be less likely to fall over and less likely to cause pain in your joints.
- If you feel at all unsteady, try using a walking stick or walking with a friend.
- Take some water with you if you are walking a long distance, especially if it is hot weather.
- If you find that you ache a lot or have body pain after doing activity, you may have done too much too quickly. Rest for a day or so and then try doing slightly less next time.
- If walking or other physical activities give you chest pain, palpitations or dizziness, or make you feel faint or fall over, you should stop doing them and tell your GP.

How can you work out your average daily step-count?

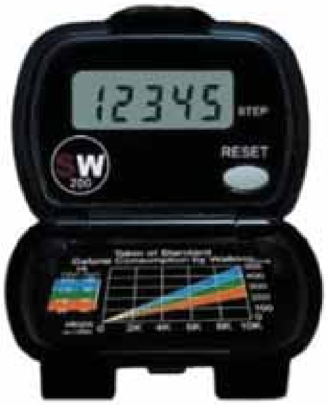

- Set the pedometer to zero before you put it on at the start of the day.
- When you take the pedometer off at night, record the number of steps in your activity diary.
- At the end of 7 days, add up the total number of steps recorded.
- Use the calculator provided to divide the total by 7.
- Just take the whole number of steps, ignore the figures after the decimal point.
- This is your average daily step-count.

Here is an example:

**Day**	**Day of the week**	**Number of steps**
1	Monday	5471
2	Tuesday	7422
3	Wednesday	6899
4	Thursday	5321
5	Friday	8072
6	Saturday	5212
7	Sunday	7419
**Total weekly steps**		**45816**
**Average daily step-count**<br><br>(total weekly steps divided by 7)		**6545**

Your personal benefits from increasing walking

What things are important to you in your life?
(*E.g. my grandchildren, my garden*)

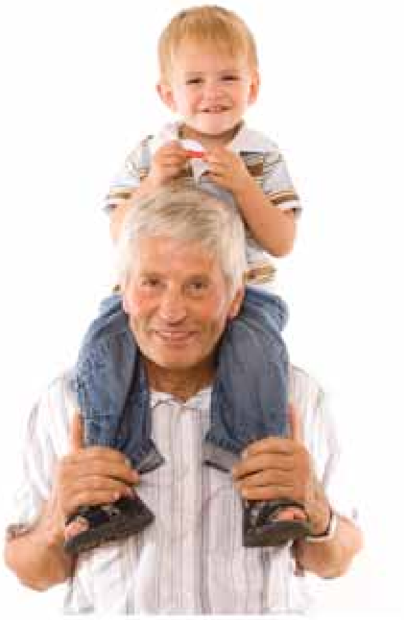

How can physical fitness help you to achieve or enjoy these things? (e.g. *I could play football with my grandson* or *I could rake the leaves in the garden*)

What other activities are important to you? Why are they important? *Some of the things that I'd like to be able to enjoy (or continue to enjoy) are:*

What are the ADVANTAGES and DISADVANTAGES of increasing your walking and activity levels?

Advantages ('Pros')	Disadvantages ('Cons')

How could you increase your daily step-count?

Make walking part of your daily routine	Ideas for new or different walks
• Walk to the shops<br>• Park at the far end of the car park<br>• Take a longer route to the library<br>• Take the stairs rather than the lift or escalator<br>• Walk to the next bus-stop before catching the bus	• Go for a stroll in your local park<br>• Walk in the countryside or woodlands<br>• Walk with family or friends<br>• With a walking group<br>• Sight-seeing, e.g. visiting a stately home or around your local town centre

How could <u>you</u> start to increase your step count? Try to include activities that are 'light' or 'moderately intense' (brisk). Ask yourself:

- How could I fit extra walking into my daily routine?
- How could I increase the number of walks that I go on?
- How could I increase the length of walks that I do?
- How can I make walking activities enjoyable and fun?

**Making walking part of my daily routine**<br>How can I build up walking during my current daily activities?	**Ideas for new or different walks**<br>Where could I walk to? Who with?<br>When would I go?

Rewards

Giving yourself little treats to reward yourself when you've made progress towards your goal can encourage you to keep going.

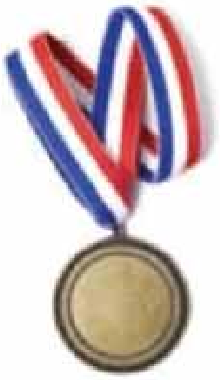

Reward yourself for any mini-goals you reach, and any other small successes that you have along the way.

Rewards don't have to cost money, but you can also 'save up' for rewards. For example, save £1 every time you do some physical activity, and at the end of the week or month, spend the money on a treat for yourself.

Try to make sure that your rewards are healthy and do not move you away from your healthy lifestyle or activity goals, such as eating food that is high in fat or sugar. Instead you could reward yourself by taking a relaxing bath, treating yourself to a new book, going to see a film or having a day out with family or friends. Or perhaps you could treat yourself to a new outfit because you have lost some weight!

How could I reward myself for achieving my goals each week?

Rewards that don't cost money	Rewards that cost money

Setting your first goals

What is my overall walking and physical activity target for the next three months?

General target:

Step count target:

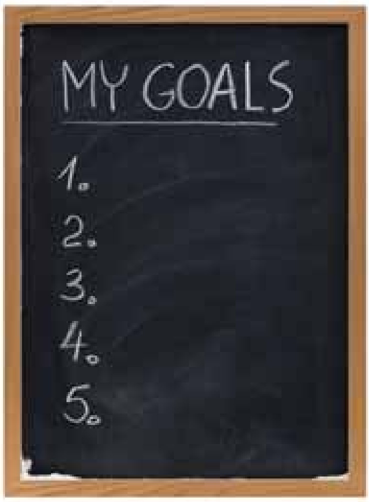

The next step is to plan some specific goals to aim for that will help to achieve your overall walking and physical activity target.

Goals need to be **SMART**: **S**pecific - **M**easurable - **A**chievable - **R**elevant – **T**imely

- **Specific** – your goal should be clear and detailed (e.g. *"I will walk my dog for at least 30 minutes three times a week, at 2pm on weekdays and 11am on Saturdays."*)
- **Measurable** – your goal should be easy to record and measure
- **Achievable** – the goal should be well within your reach
- **Relevant** – try to keep in mind why walking is important for you
- **Timely** – plan a realistic time to try to achieve your goal

Larger goals can be broken down into smaller steps or 'mini-goals' so that you can achieve something each week. For example:

**Where I am now**	**STEP 1** Week one	**STEP 2** Week two	**STEP 3** Week three	**STEP 4** Week four
A friend gives me a lift to the shop to buy my paper	Walk to the shop and get a lift back	Walk to the shop and back and time myself	Walk to the shop and back and time myself to take 15 minutes each way	Walk to the shop and back taking 10 minutes each way

SMART Steps to my target

The next step is to set some SMART walking goals to aim for before your next meeting with the Physical Activity nurse.

Many people find that trying to **increase their steps by 10% of their current walking levels** is a realistic target to aim for in the first couple of weeks. Do you think you might be able to achieve this?

You could use the Walking Planner which is located in section 1 of this physical activity folder to plan some walking goals.

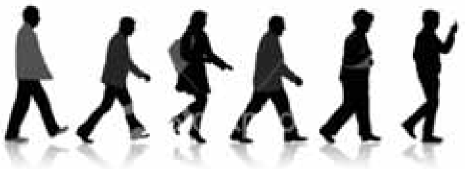

When setting your goals, try to ask yourself:

- **What** am I going to do?
- **Where** am I going to do it?
- **When** am I going to do it?
- **Who** am I going to do it with?
- **How long** am I going to do it for?

How will I know how I'm doing?

It is important to measure and record your progress, so you can be motivated when you see that you are succeeding, and to work out what you can change if your plan is not working.

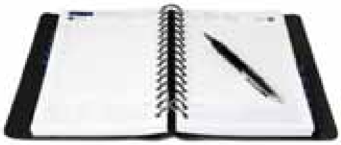

You might find it helpful to take a look at the **Physical Activity and Step-Count Diary,** which is located in section 6 of this physical activity folder, and is designed to help keep track of your walking each week.

What will I record in my diary?

....

When will I record it?

....

Where will I keep my diary?

How confident are you to achieve your goals?

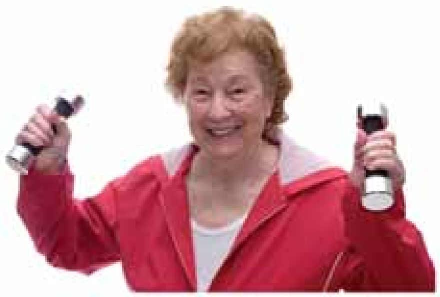

People with a **high** level of confidence in their ability to change are more likely to achieve their goals and are more likely to solve problems that get in their way.

Measuring your confidence level with a confidence ruler

On a scale of 1 to 10, how confident do you feel that you can achieve this goal (if 1 is not confident and 10 is very confident)?

Not confident 1-----2-----3-----4-----5-----6-----7-----8-----9-----10 Very confident

If your score is greater than 7: Excellent – you are confident at achieving this goal!

If your score is less than 7: You may need to set a more realistic goal. How could you break your goal down into smaller steps?

**Where am I now?**	**STEP 1** By the next time I see the nurse...	**STEP 2**	**STEP 3**	**STEP 4**	**Where would I like to be?** In 3 months...
**How many steps?**	e.g. *my steps have gone up from 5000 a day to 5500 a day*				
**What am I doing to achieve this?**	*I am walking the long route to the shops every day*				

Final checklist for session 1

- Do I know **what** I am planning to do and **when**?
- Do I feel **confident** that I can achieve these goals (confidence ruler)
  If not, do I need to work on my **confidence** levels or remind myself of its **importance** to me, before tackling the goals?
- Do I know what **rewards** I will give myself and when for achieving my goals?
- Do I have a **plan** for reviewing and setting my next set of goals (e.g. after one week)?
- Is there **anything else** that I would like to discuss with the nurse?

Date and time of next meeting..

(Please bring along your folder and the record of your step-counts to your next meeting)

Before our next meeting I will wear the accelerometer for 7 days from

....

Session Two – Continuing the changes

Reviewing your last goals

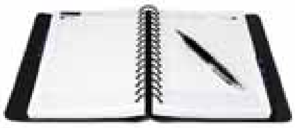

The first step is to review what happened since you last saw the Physical Activity nurse and how you got on with the goals you set last time. **Remember to praise yourself for any success, no matter how small it seems!**

Why not start by turning to your **Physical Activity and Step-count diary** and discussing your activity levels for the past week with your Physical Activity nurse? Then turn to the last session's **Walking Planner** and complete the '**Reviewing Goals: What happened?**' section. Ask yourself:

- What did you try (and what was the goal)?
- Did you find it easy or difficult to achieve? Was it a realistic goal?
- What was the impact of the behaviour? What benefits were there?
- What difficulties were there? How did you manage these?
- What have you learned that could be useful when planning future goals?
- Did you remember to reward yourself for making any positive changes?
- Are you getting enough support?
- Did you have any problems using the equipment?
- Were there any problems filling in the Physical Activity Diary [see box below]?

Did you have any problems with using the Physical Activity and Step Count Diary?

- What is made it difficult for you to use your diary?
- Did you have enough time?
- How do you feel about writing things down?
- Can you see the benefits of completing the diary? What can the diary tell you?
- Can you get family or friends to help you fill it in?
- Can you think of any other solutions to help you complete it?

Barriers to increasing my physical activity

Barriers and obstacles to change are things that make it difficult for us to make changes or to stick to them over time. Can you think of any ways to overcome these difficulties and make the changes that are important to you?

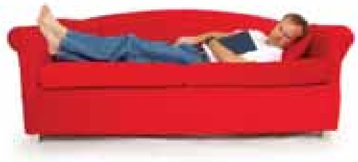	**Places and things:** Do any particular places or things around me make it especially difficult to increase my physical activity? How could I get around this problem?
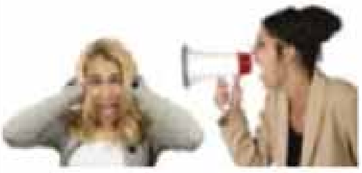	**People:** Are there any people I spend time with who make it difficult to increase my physical activity? What can I do to overcome this?
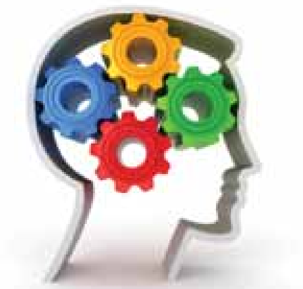	**Thoughts and feelings**: Do any particular thoughts, attitudes or emotions make it difficult to do this? How could I overcome these?
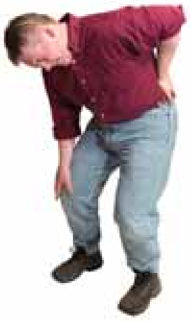	**Physical symptoms and reactions**: Do any physical symptoms or body reactions make it difficult to be more active? How could I safely overcome these?

Facilitators to increasing physical activity

Facilitators to change are things that make it easier to make the changes that we want to. Thinking about these will help to make change more possible.

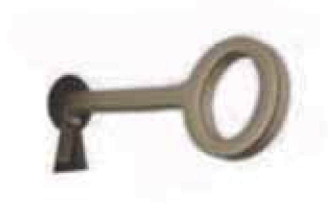	**Places and things:** Is there anything about my surroundings that makes it easier to increase my physical activity? What can I do to use these helpful things?
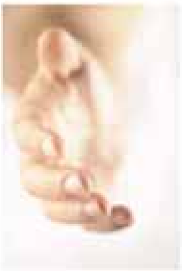	**People:** Are there any people I spend time with who make it easier to increase physical activity? How could they help me?
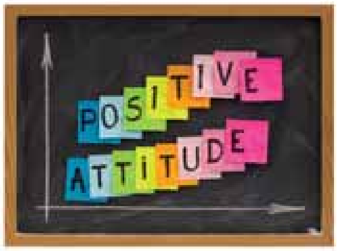	**Thoughts and feelings:** Do any thoughts, attitudes or emotions make it easier to walk more? How can I encourage these?
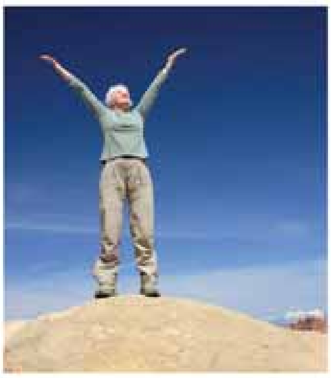	**Physical symptoms and reactions:** What are the physical benefits of increasing my walking? E.g. feeling more energetic, clothes looser or having more stamina.

Overcoming barriers to change

Keeping your 'facilitators to change' in mind, can you think of any ways to overcome the obstacles that prevent you from achieving your last set of goals? You may need to experiment and try several solutions before you find one that works well.

Barrier or obstacle	Ideas for solution(s) to overcome this difficulty	Which solution will I try first and when?
	**1.**<br><br>**2.**<br><br>**3.**	
	**1.**<br><br>**2.**<br><br>**3.**	
	**1.**<br><br>**2.**<br><br>**3.**	
	**1.**<br><br>**2.**<br><br>**3.**	

Goal setting

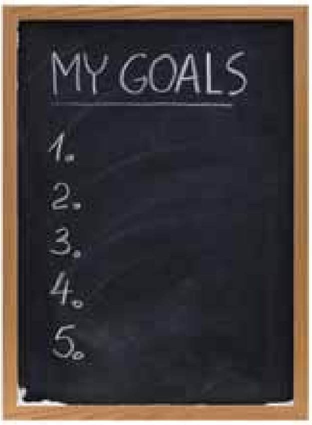

This is the stage in which you can set some more physical activity goals to aim for before the next meeting with your Physical Activity nurse.

STEP ONE: Review your Physical Activity Target for the next 3 months.
Is this target still realistic and does it fit with what is important to you?

STEP TWO: Set some new SMART goals and record them in your Weekly Walking Planner
Can you build on any success that you had last time?
Can you include goals for...?

- Your overall step-count for the next week (are you able to aim for an **increase in steps of 10%** from last time?)
- Goals for increasing walking within your daily routine
- Goals for new and different walking activities

STEP THREE: Think about any barriers or obstacles that might get in the way
Include in your plan how you might overcome these difficulties

STEP FOUR: Check your confidence using a Confidence Ruler

- If you are not confident to achieve these goals, how could you increase this (see optional confidence building section below)?
- Do you need to set more realistic goals or break bigger targets down into 'mini-goals'?

SMART goals reminder: **S**pecific - **M**easurable - **A**chievable - **R**elevant – **T**imely

• **What** am I going to do?	**Where** am I going to do it?
• **When** am I going to do it?	**Who** am I going to do it with?
• **How long** am I going to do it for?	**How** will I reward myself?
• **What** will I record in my diary?	**When** will I record it?

Building on your confidence to change (optional)

Measuring your confidence level with a confidence ruler

How confident do you feel that you can achieve this goal? (if 1 is not confident and 10 is very confident)

Not confident 1-----2-----3-----4-----5-----6-----7-----8-----9-----10 Very confident

If your score is greater than 7: Excellent – you are confident at achieving this goal!

If your score is less than 7: You may need to set a more realistic goal or to build on your confidence using the exercises below.

If you have low confidence then you could try asking some of the following questions to help to boost your confidence to achieve your goals:

Why not an even lower number? What gives you confidence already? How could you build on this and move up the scale?

What successes and achievements have you made in the past? How did you achieve these? What attitudes did you have? What actions did you take? How did you overcome any problems? Can this boost your confidence to make change?

Can I get any inspiration or support from others? Do you know of anyone (e.g. a friend, family member, neighbour) who has been successful in changing something in their life? How did they go about this? How could friends and family support or encourage you?

What would you do differently if you felt more confident? Can you do some of these things anyway? By behaving **'as if'** you feel more confident then you may start to feel the same way.

Reviewing and setting your own goals (optional)

Your next meeting with the Physical Activity nurse will be in four weeks time and you can stick with the goals that you have planned today for this entire time.

However, if you are feeling ambitious, you could plan to review and set some more goals yourself before your next session with the nurse (e.g. after two weeks). You could add this onto your weekly Goal Planning chart as a reminder.

When reviewing and setting your goals, you might find the following checklist helpful. Ask yourself:

- What did you try (and what was the goal)?
- What was the impact of the behaviour? What benefits were there?
- Did you find it easy or difficult to achieve? Was it a realistic goal?
- What difficulties, barriers or obstacles got in the way of achieving your goals? How did you manage these? How might you overcome them in future?
- Are you getting enough support?
- What have you learned that could be useful when planning future goals?
- Did you remember to reward yourself for making any positive changes?
- How could you build on your success from the past week?
- What SMART goals do you plan to carry out over the next week? (Complete this in a new goal planning chart).

Final checklist for Session Two

- Do I know **what** I am planning to do and **when**?
- Do I feel **confident** that I can achieve these goals (confidence ruler)
  If not, do I need to work on my **confidence** levels or remind myself of its **importance** to me, before tackling the goals?
- Do I know what **rewards** I will give myself and when for achieving my goals?
- Do I have a **plan** for reviewing and setting my next set of goals (e.g. after one week)?
- Is there **anything else** that I would like to discuss with the nurse?

Date and time of next meeting...

(Please bring along your folder and the record of your step-counts to your next meeting)

Before our next meeting I will wear the accelerometer for 7 days from

....

Session Three: Keeping up the changes

Reviewing your last goals

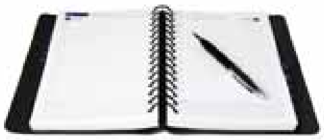

As usual, the first step is to review what happened since you last saw the Physical Activity nurse and how you got on with the goals you set last time. You may have also managed to set your own goals between sessions and you can also discuss how this went.

Why not start by turning to your **Physical Activity and Step-count diary** and talking through your activity levels for the past week.

Then turn to last week's **Walking Planner** and complete the **'Reviewing Goals: What happened?'** section. Try to keep in mind the following:

- **Key questions for reviewing your goals**
- **Problem-solving and overcoming any barriers**
- **Give yourself praise and rewards!**
- **If you set your own goals, what helped you do this?**

Make a note here of anything that you have learned from this discussion:

Preparing for setbacks

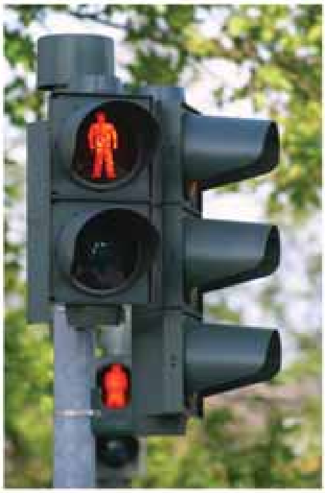

Changing behaviour is the first step; the next is to maintain that change. Successful change is not a smooth process; it doesn't go in a straight line.

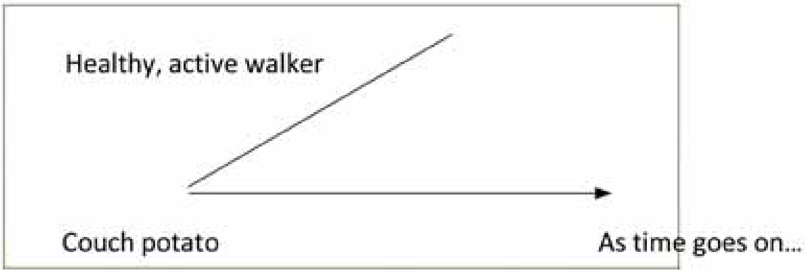

Successful change usually involves ups and downs. You will not be instantly transformed into a regular, active walker and will probably experience plateaus and setbacks at times, before things pick up and improve once more.

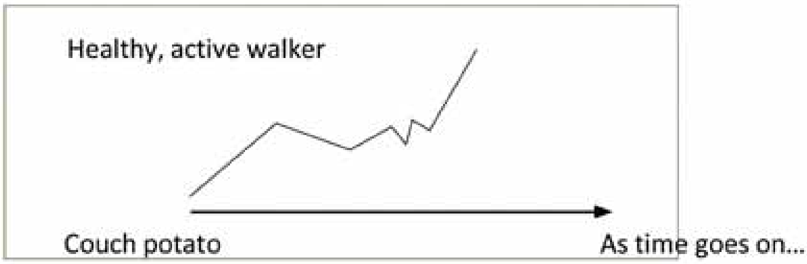

It is important not to get disheartened when you experience a setback, but to see it as an opportunity for learning. Key points to prepare for setbacks:

1. **Avoid 'risky' situations**, for example, sitting down and watching TV ten minutes before you need to leave for a planned walk

2. **Develop ways of 'coping'** when you find yourself in a 'high-risk' situation, for example, carry a banana in your bag to eat if you get hungry whilst out walking.

How could you plan to cope with any setbacks?
There are some ideas and tips in an optional background reading section *(called "Dealing with Setbacks")* at the end of this session.

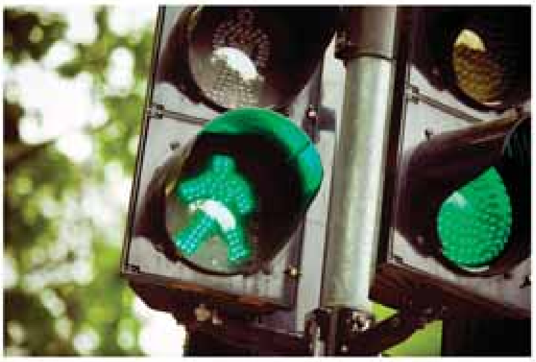

Pacing – avoiding the 'boom-bust' trap

Many people believe that they should only be active on 'good' days when they feel 100% fit and well, but this is not necessarily the case. Gentle activity can have many benefits including increasing energy levels, reducing lethargy and tiredness and improving your mood. Equally, it is often better not to *over-do* things and try to do too much on a 'good' day where you feel fit and energetic, as this may make you at risk of injury and fatigue, which results in a gradual reduction in your fitness over time.

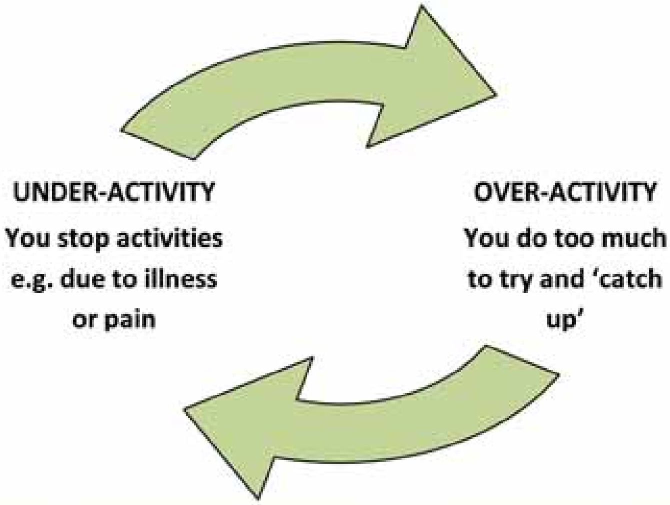

Example – Boom and bust: Mary had been suffering from a cold but she was also keen to buy her grand-daughter a birthday present. She decided to rest for a week until over her cold, and as soon as she felt better, she went to the shopping centre and spent all day walking around the shops. Afterwards she felt exhausted with achy muscles. She needed to rest again for several days.

Planning activities using pacing

Rather than stopping your activities altogether when feeling tired or unwell, it is better to try to reduce your activity to a planned base level, from which you can then gradually build back up when feeling better. This process is known as **pacing** and it can help to build up fitness steadily over time.

Example – Pacing: Mary rested for the first two days of her cold and then re-started building up her walking gradually in the garden and to the local shop. Then, rather than trying to spend all day at the shopping centre, she took the bus to the local town centre for an hour and was able to find a present for her grand-daughter. She felt well after this and was able to continue being active.

Pacing tips

- Stop **before** you get tired (don't carry on until you are exhausted)
- Alternate heavier physical exercise with more moderate or gentle activity on the following day
- Try to balance activities across the week
- Start slowly and build up gradually. Schedule in rest breaks if needed.
- Break the activity down into smaller stages (e.g. 10 minutes at a time) if you need to
- Avoid vigorous activity if you are unwell, injured or fatigued

Your own pacing tips and plans: How do you plan to cope with illness, injuries or setbacks? How can you avoid the 'boom-bust' trap?

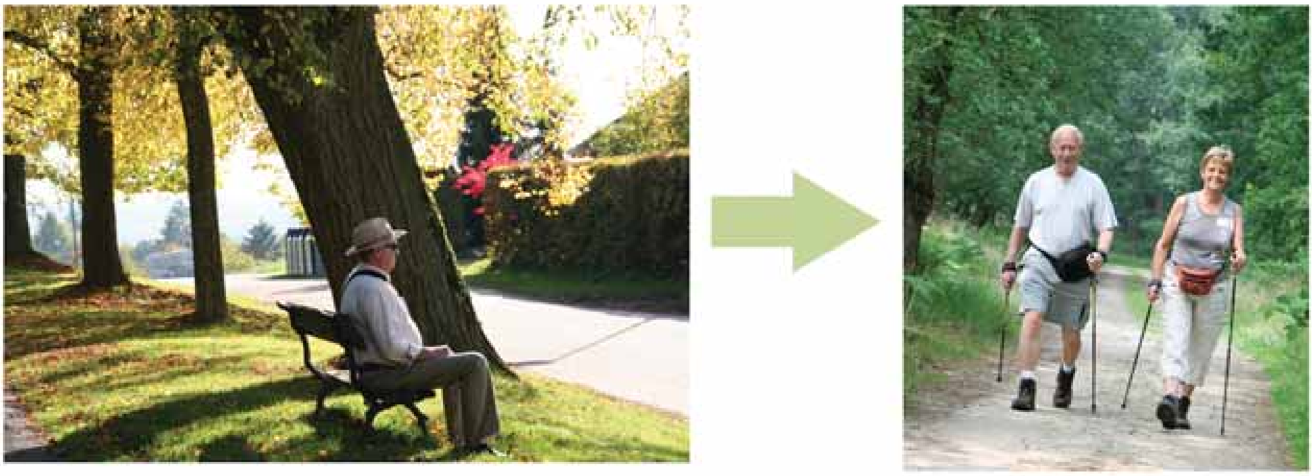

Keeping safe: If walking or other physical activities are giving you chest pain, palpitations or dizziness, or making you feel faint or fall over, you should always stop doing them and tell your GP.

Building habits: 'if-then' plans

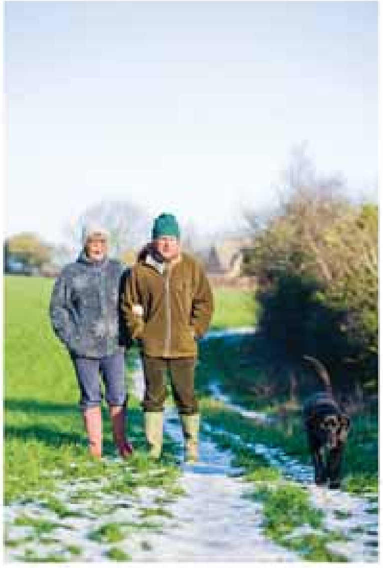

Another way of coping with potential difficulties is to devise an 'if-then' plan. This requires you to specify exactly what you are going to do and when and the situation you are going to do it in.

If-then plans can be helpful to **prevent setbacks**, e.g.:

- **If** I feel tempted to go to the pub instead of going walking **then** I will ring my friend and ask him to come with me

Or they can be used for **pacing**:

- **If** I am feeling very tired, **then** I will try a gentle 10 minute walk rather than putting it off completely

Or they can be used to **build good habits**, e.g.:

- **If** I am going to the shopping centre, **then** I will walk up the stairs instead of taking the lift

In the chart below, make a list of some 'if-then' rules that could help you to stick to your activity goals.

What is the situation?	What will I do if it arises?
If...	Then....
If...	Then....
If...	Then....
If...	Then....
If...	Then....

Goal setting

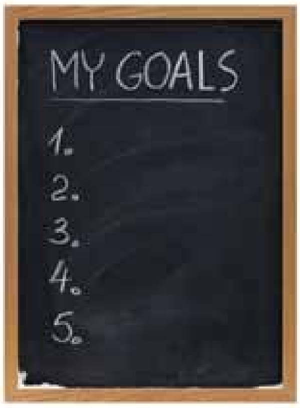

This is the stage in which you can set some more physical activity goals to aim for before the next meeting with your Physical Activity nurse.

STEP ONE: Review your Physical Activity Target for the next 3 months.

Is this target still realistic and does it fit with what is important to you?

STEP TWO: Set some new SMART goals and record them in your Weekly Walking Planner

Can you build on any success that you had last time?

Can you include goals for...?

- Your overall step-count for the next week (are you able to aim for an **increase in steps of 10%** from last time?)
- Goals for increasing walking within your daily routine
- Goals for new and different walking activities

STEP THREE: Think about any barriers or obstacles that might get in the way

Include in your plan how you might overcome these difficulties

STEP FOUR: Check your confidence using a Confidence Ruler

- If you are not confident to achieve these goals, how could you increase this (see optional confidence building section below)?
- Do you need to set more realistic goals or break bigger targets down into 'mini-goals'?

SMART goals reminder: **S**pecific - **M**easurable - **A**chievable - **R**elevant – **T**imely

- **What** am I going to do? **Where** am I going to do it?
- **When** am I going to do it? **Who** am I going to do it with?
- **How long** am I going to do it for? **How** will I reward myself?
- **What** will I record in my diary? **When** will I record it?

Reviewing and setting your own goals (<u>optional</u>)

Your next meeting with the Physical Activity nurse will be in four weeks time. You could stick with the goals that you have planned today for this entire time, or if you are feeling ambitious, you could plan to review and set some more goals yourself before your next session with the nurse (e.g. after two weeks).

If you would like to set some more goals yourself, you may wish to add this onto your weekly Goal Planning chart as a reminder.

When reviewing and setting your goals, you might find the following checklist helpful. Ask yourself:

- What did you try (and what was the goal)?
- What was the impact of the behaviour? What benefits were there?
- Did you find it easy or difficult to achieve? Was it a realistic goal?
- What difficulties, barriers or obstacles got in the way of achieving your goals? How did you manage these? How might you overcome them in future?
- Are you getting enough support?
- What have you learned that could be useful when planning future goals?
- Did you remember to reward yourself for making any positive changes?
- How could you build on your success from the past week?
- What SMART goals do you plan to carry out over the next week? (Complete this in a new goal planning chart).

Dealing with setbacks (optional background reading)

Everyone makes mistakes!

Don't be too hard on yourself - it's not the end of the world! Missing a few walks is not failure, unless you let it become one. It is fine to drop back to an earlier stage and start building up again from there. The important thing is not to give up, but to keep on trying.

Are you still recording your walking activities and daily step-counts?

If you have stopped recording how often you do or don't do your walks or keep a record of your step-counts for each day, it's easy not to realise that you're slipping away from your goals.

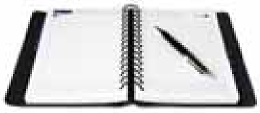

What is making it difficult for you to record this? Can you think of anything to make it easier?

Coping with difficult situations and triggers

Are there certain **times**, **places**, or **people** in whose company you feel the temptation to slip back to your old habits? It might help you to look back at the list of **barriers and obstacles to change** and your action plan for overcoming these difficulties.

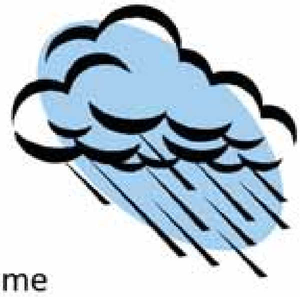

Have any of these situations changed? Are there new obstacles? Do some coping plans work better than others?

Are you remembering to give yourself rewards?

Giving yourself little treats to reward yourself when you've made progress can encourage you to keep going. Make a big deal of it when you achieve any mini-goals; you've worked hard, you deserve it!

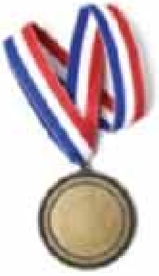

Remember, rewards don't have to cost money. Why not think of some new treats to add to your list!

Support – are you getting enough?

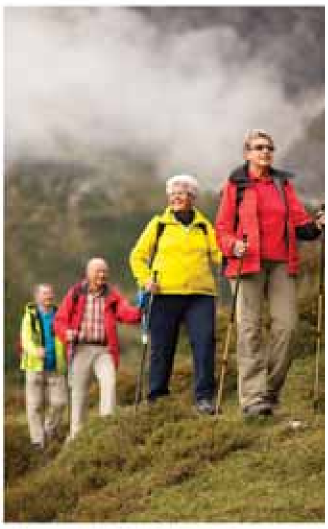

Are you getting enough support from your friends, family and your community with increasing your walking? If the answer is **no**, think about the following questions:

- Are there certain people who are good at helping me to keep up my walking? If yes, can I spend more time with them / speak to them more often?
- How can the community help? Do you have any local walking groups? Does your local library, sports or health centre have any information that you need? Could you gain any information or support via the internet?

Remind yourself why the changes are important to you

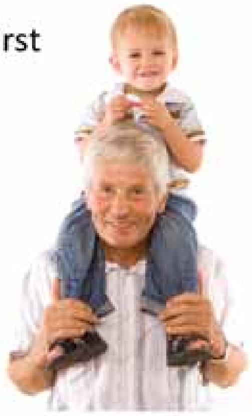

Try not to forget why you committed to making these changes in the first place. Remind yourself of the reasons that it is important for **<u>you</u>** to increase your activity and fitness levels. For example:

- *"Because I want to stay mobile and independent for as long as possible"*
- *"Because keeping in shape makes me feel good about myself"*
- *"Because it will help me keep my heart healthy"*

What are <u>your</u> main reasons for keeping fit and active?

Final checklist for session 3

- Do I know **what** I am planning to do and **when**?
- Do I feel **confident** that I can achieve these goals (confidence ruler)
  If not, do I need to work on my **confidence** levels or remind myself of its **importance** to me, before tackling the goals?
- Do I know what **rewards** I will give myself and when for achieving my goals?
- Do I have a **plan** for reviewing and setting my next set of goals (e.g. after one week)?
- Is there **anything else** that I would like to discuss with the nurse?

Date and time of next meeting...

(Please bring along your folder and the record of your step-counts to your next meeting)

Before our next meeting I will wear the accelerometer for 7 days from

....

Session Four: Building lasting habits

This is your final session with the Physical Activity nurse. **Congratulations on your progress so far!**

How did you get on with the goals you set last time? As usual, the first step is to review how you got on since you last saw the Physical Activity nurse, using your **Physical Activity and Step-count diary** and last session's **Walking Planner** as reminders.

Reviewing your progress

At this stage it is often helpful to remind yourself of the progress that you have made so far. Why not look back through some of your earlier Physical Activity and Step-count diary sheets to review what changes you have made. Ask yourself:

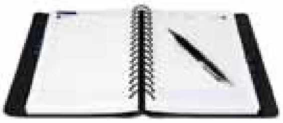

What changes have I made in my daily and weekly step-counts?

What am I doing differently now? How have my activities changed?

What are the main benefits of the walking programme that I have noticed?

Building habits

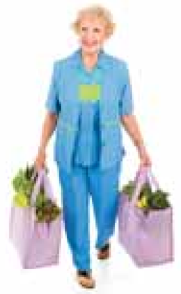

Once you have successfully increased your walking and step-count, you need to maintain it and continue into the foreseeable future. It needs to become part of your lifestyle, an automatic habit that doesn't require effort or thinking.

Based on your own experience of the walking programme over the past weeks, what advice could you give yourself on how to keep up the changes you have made?

Other ways of building habits include:

'If-then' rules

These can be used to help to remind you to do a particular activity. For example, *"**If** it is a weekday morning, **then** I will get up by 8am and walk to the shop to buy a paper"*

Repetition

Simply by repeating their new behaviour over and over it is more likely to become a habit and will be easier to carry it out in future.

Support

Are you continuing to use the support you can get from friends, family and the community? Positive support and encouragement can help to keep going with any new behaviour.

Rewards

Remember to rewards yourself for achieving goals and mini-goals; this will encourage you to keep up your new activity habits.

Reviewing progress, benefits and outcomes

Remember to look back at what you have already achieved. This will help build your confidence to continue to make changes.

Action Stations - Becoming your own physical activity coach

This next section will help you to create an action plan for keeping up the changes and continuing to build up your walking and step-count. It contains a reminder of all the techniques that you have used so far, which you can use in future to help you continue to make progress.

My personal benefits for maintaining or increasing physical activity

The things that make it most important for me to maintain progress in my walking activity and step-count are:

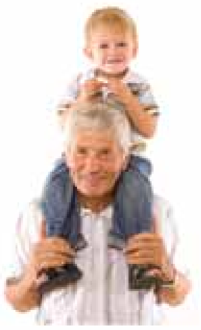

What are the advantages and disadvantages of making the change?

If I DO change my behaviour	
Advantages	Disadvantages

If I DON'T change my behaviour	
Advantages	Disadvantages

Setting a new overall activity target

What is my overall exercise and activity target for the next three months?

General target:

Step count target:

How can I achieve my activity target?

Which specific activities can you plan to continue or further build on over the next three months that will help to achieve your new activity target? Try to think about:

- Daily or weekly step-count targets (you could try to increase by 10% each time)
- Ideas for including walking within your daily routine
- Ideas for maintaining or increasing the number and length of walks that you take
- Ideas for making walking activities enjoyable and fun

Make your goals are SMART: **S**pecific - **M**easurable - **A**chievable – **R**elevant - **T**imely

Remember, you can use a Walking Planner to help plan your walking goals!

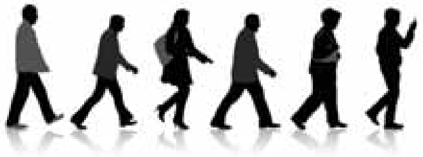

PLANNING WALKING GOALS		REVIEWING GOALS: WHAT HAPPENED?	
**When?**	**What do I plan to do? (SMART goals)**<br>What? Where? Who with? How long for?<br>What will be my reward?	**Did I do this?**<br>Yes No Partly	**Comments**<br>What made it easy or difficult?<br>How did I feel after? (emotionally / physically)

Remember to give yourself rewards

Which are your favourites?

Rewards that cost money	Rewards that don't cost money

Confidence check

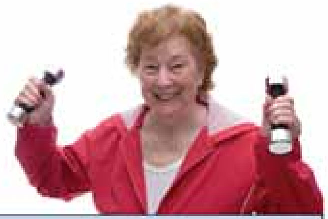

How confident are you that you will be able to achieve your goals?
Use the confidence ruler to find out...

Measuring your confidence level with a confidence ruler

How confident do you feel that you can achieve this goal (if 1 is not confident and 10 is very confident)?

Not confident 1-----2-----3-----4-----5-----6-----7-----8-----9-----10 Very confident

If your score is greater than 7: Excellent – you are confident at achieving this goal!

If your score is less than 7: You may need to set a more realistic goal or to work on building your confidence

If your confidence is low...

- Think about your own past successes and achievements
- Look for ideas from other people you respect or admire
- Get support from friends, family and your community
- Try breaking your target down into mini-goals

**Where I am now**	**STEP 1** E.g. in a week's time	**STEP 2**	**STEP 3**	**STEP 4**	**Where would I like to be?** In 3 months...

Do it! Go walking!

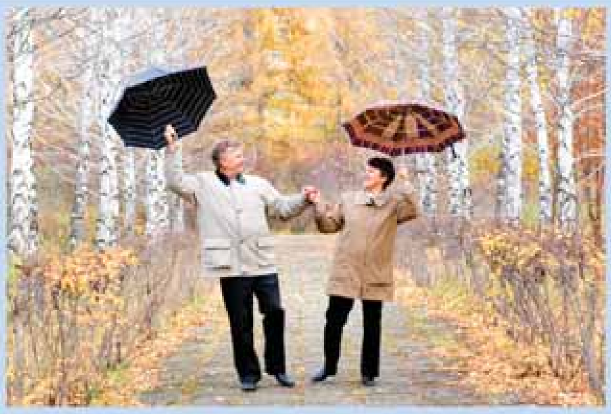

Record it... Reward it... Repeat it... Review it....

- Record your **goal** for the week(s) in your diary sheet
- Record your **activity** and also note how you **felt** about it
- Don't forget to **congratulate** and **reward** yourself
- **Repeat** your physical activity – keep it up!
- **Review** what happened: look at the following table for ideas to review your goals

Review your goals

Successful	Partly successful or less successful
• Congratulate yourself – what were the benefits of this?<br><br>Next steps:<br>• Try the same goal again<br>• Or set yourself a more difficult SMART goal	• What was different on days when you made some progress?<br>• What made it difficult or got in the way?<br>• Was it a realistic goal?<br>• How can you make it easier? (problem-solving)<br>• Try again or set an easier SMART goal

Setting new goals

It is also important to set yourself new goals. Many people find it helpful to do this each week or month. When would be a good time for you to review and set your new goals?

Overcoming barriers and obstacles

Can you think of any ways to overcome any difficulties and make the changes which are important to you?

E.g. If it is raining then I will put on a raincoat and take an umbrella!

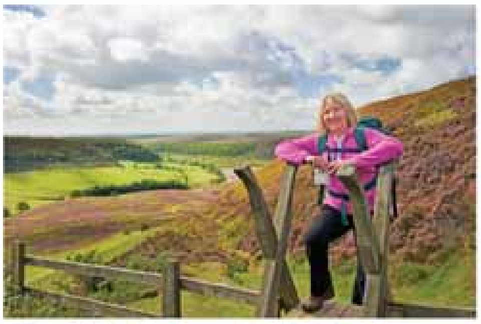

Barrier or obstacle	Ideas for solution(s) to overcome this difficulty	Which solution will I try first and when?

Pacing and avoiding the boom-bust trap

Ensure that you build up your walking levels steadily and that you avoid the 'boom-bust' trap of over and under-activity.

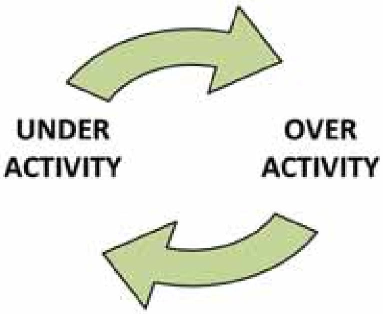

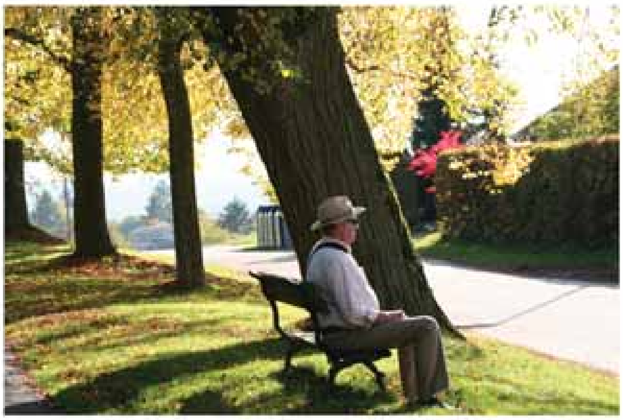

What steps you can take to include 'pacing' in your activity plan (see next page)?

Ask yourself...

How will I react when I am feeling tired or unwell?

What will be my <u>minimum</u> walking activity level to aim for?

Dealing with setbacks and maintaining good habits

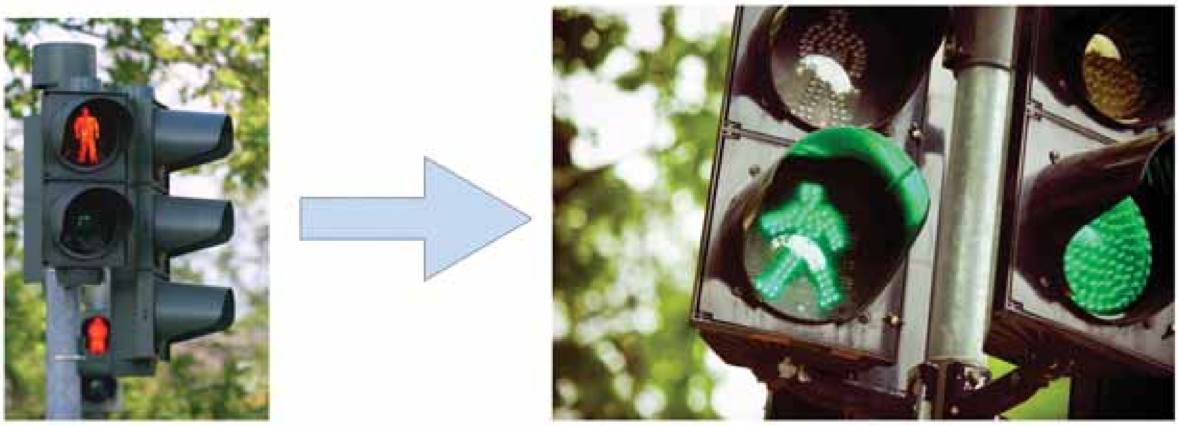

Try creating some more **'if-then' action plans** for dealing with setbacks

If ....happens	Then I will..
If...	Then I will...
If...	Then I will...

The most useful steps of this plan that will keep me going are:

....

....

....

....

....

Congratulations!

Keep Going!

The PACE-Lift research assistant will contact you shortly after your last nurse visit. She will arrange with you a time to check your physical activity levels.
She will contact you again, a year after you started seeing the nurse, to see how you are progressing

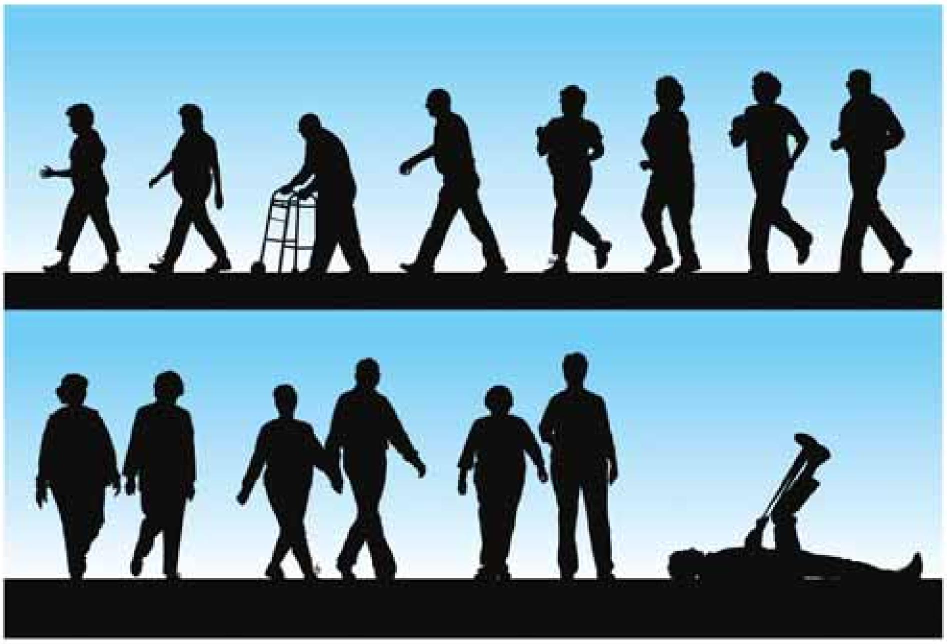

Walking planner **Date:**

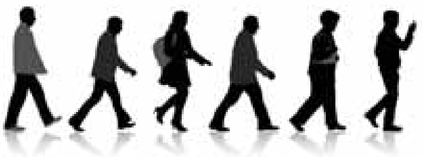

Reminder: My overall three month walking target is...

Now set some walking targets for before your next meeting with the Physical Activity Nurse:

Daily Step-Count goal (how many steps to aim for every day) ________

Weekly Step-Count goal (how many steps to aim for every week) _________

How can I achieve my walking and step-count goals? Complete the "planning walking goals" section of the following table.

PLANNING WALKING GOALS		REVIEWING GOALS: WHAT HAPPENED?	
**When?**	**What do I plan to do? (SMART goals)**<br>What? Where? Who with? How long for?<br>What will be my reward?	**Did I do this?**<br>Yes No Partly	**Comments**<br>What made it easy or difficult?<br>How did I feel after? (emotionally / physically)

Walking planner **Date:**

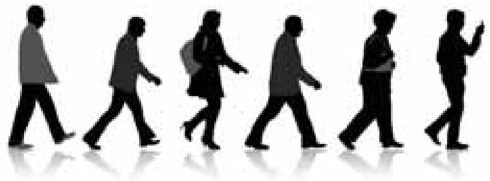

Reminder: My overall three month walking target is...

Now set some walking targets for before your next meeting with the Physical Activity Nurse:

Daily Step-Count goal (how many steps to aim for every day) ________

Weekly Step-Count goal (how many steps to aim for every week) _________

How can I achieve my walking and step-count goals? Complete the "planning walking goals" section of the following table.

PLANNING WALKING GOALS		REVIEWING GOALS: WHAT HAPPENED?	
**When?**	**What do I plan to do? (SMART goals)**<br>What? Where? Who with? How long for?<br>What will be my reward?	**Did I do this?**<br>Yes No Partly	**Comments**<br>What made it easy or difficult?<br>How did I feel after? (emotionally / physically)

Walking planner **Date:**

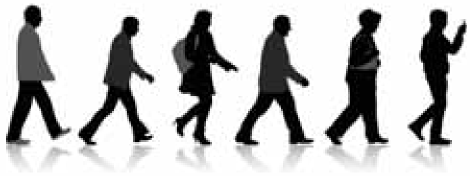

Reminder: My overall three month walking target is...

Now set some walking targets for before your next meeting with the Physical Activity Nurse:

Daily Step-Count goal (how many steps to aim for every day) ________

Weekly Step-Count goal (how many steps to aim for every week) _________

How can I achieve my walking and step-count goals? Complete the "planning walking goals" section of the following table.

PLANNING WALKING GOALS		REVIEWING GOALS: WHAT HAPPENED?	
**When?**	**What do I plan to do? (SMART goals)**<br>What? Where? Who with? How long for?<br>What will be my reward?	**Did I do this?**<br>Yes No Partly	**Comments**<br>What made it easy or difficult?<br>How did I feel after? (emotionally / physically)

Walking planner **Date:**

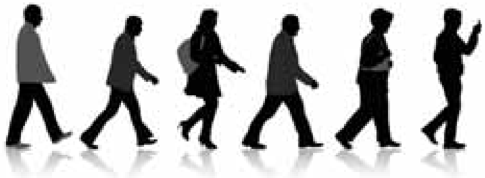

Reminder: My overall three month walking target is...

Now set some walking targets for before your next meeting with the Physical Activity Nurse:

Daily Step-Count goal (how many steps to aim for every day) ________

Weekly Step-Count goal (how many steps to aim for every week) _________

How can I achieve my walking and step-count goals? Complete the "planning walking goals" section of the following table.

PLANNING WALKING GOALS		REVIEWING GOALS: WHAT HAPPENED?	
**When?**	**What do I plan to do? (SMART goals)**<br>What? Where? Who with? How long for?<br>What will be my reward?	**Did I do this?**<br>Yes No Partly	**Comments**<br>What made it easy or difficult?<br>How did I feel after? (emotionally / physically)
