# Supplementary material for: A Primary Care Nurse-Delivered Walking Intervention in Older Adults: PACE (Pedometer Accelerometer Consultation Evaluation)-Lift Cluster Randomised Controlled Trial
Source: PLoS Med. 2015 Feb 17;12(2):e1001783. doi: 10.1371/journal.pmed.1001783 (PMC4331517; doi:10.1371/journal.pmed.1001783)
Supplement: S4 Text — (PDF) [file pmed.1001783.s010.pdf]

Physical Activity and Step-Count Diary **Week 1** **Daily Step-Count goal ________**

Weekly Step-Count goal _________

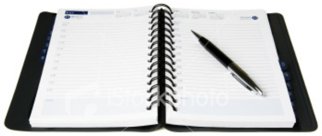

Use this diary to record any physical activities you do this week. This includes **walking** and **other physical activities** (e.g. cycling gardening, swimming, heavy housework, exercise classes etc). Write down the **approximate time** and **how long** you do them for.

**Date**							
**Day of week**							
**Morning**							
**Afternoon**							
**Evening**							
**Daily step-count**							

Add up the steps from all 7 days to get total weekly step-count: **Total weekly step-count = ________**

Divide the total weekly step-count by 7 to get average daily step-count: **Average daily step-count = ________**

Did I achieve my goal? **Yes: And my reward was**……………………………………………………………………………………………………….................

No / Partly: What got in the way? ....

Physical Activity and Step-Count Diary **Week 2** **Daily Step-Count goal ________**

Weekly Step-Count goal _________

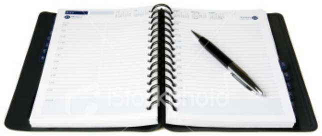

Use this diary to record any physical activities you do this week. This includes **walking** and **other physical activities** (e.g. cycling gardening, swimming, heavy housework, exercise classes etc). Write down the **approximate time** and **how long** you do them for.

**Date**							
**Day of week**							
**Morning**							
**Afternoon**							
**Evening**							
**Daily step-count**							

Add up the steps from all 7 days to get total weekly step-count: **Total weekly step-count = ________**

Divide the total weekly step-count by 7 to get average daily step-count: **Average daily step-count = ________**

Did I achieve my goal? **Yes: And my reward was**...

No / Partly: What got in the way? ....

Physical Activity and Step-Count Diary **Week 3** **Daily Step-Count goal ________**

Weekly Step-Count goal _________

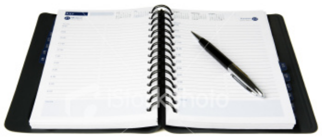

Use this diary to record any physical activities you do this week. This includes **walking** and **other physical activities** (e.g. cycling gardening, swimming, heavy housework, exercise classes etc). Write down the **approximate time** and **how long** you do them for.

**Date**							
**Day of week**							
**Morning**							
**Afternoon**							
**Evening**							
**Daily step-count**							

Add up the steps from all 7 days to get total weekly step-count: **Total weekly step-count = ________**

Divide the total weekly step-count by 7 to get average daily step-count: **Average daily step-count = ________**

Did I achieve my goal? **Yes: And my reward was**………....................

No / Partly: What got in the way? ....

Physical Activity and Step-Count Diary

Week 4 **Daily Step-Count goal ________**

Weekly Step-Count goal _________

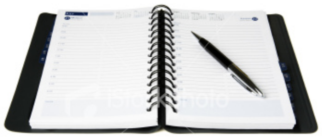

Use this diary to record any physical activities you do this week. This includes **walking** and **other physical activities** (e.g. cycling gardening, swimming, heavy housework, exercise classes etc). Write down the **approximate time** and **how long** you do them for.

**Date**							
**Day of week**							
**Morning**							
**Afternoon**							
**Evening**							
**Daily step-count**							

Add up the steps from all 7 days to get total weekly step-count: **Total weekly step-count = ________**

Divide the total weekly step-count by 7 to get average daily step-count: **Average daily step-count = ________**

Did I achieve my goal? **Yes: And my reward was**……

No / Partly: What got in the way? ………

Physical Activity and Step-Count Diary

Week 5 **Daily Step-Count goal ________**

Weekly Step-Count goal _________

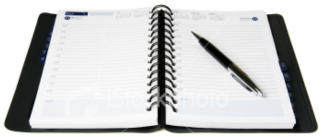

Use this diary to record any physical activities you do this week. This includes **walking** and **other physical activities** (e.g. cycling gardening, swimming, heavy housework, exercise classes etc). Write down the **approximate time** and **how long** you do them for.

**Date**							
**Day of week**							
**Morning**							
**Afternoon**							
**Evening**							
**Daily step-count**							

Add up the steps from all 7 days to get total weekly step-count: **Total weekly step-count = ________**

Divide the total weekly step-count by 7 to get average daily step-count: **Average daily step-count = ________**

Did I achieve my goal? **Yes: And my reward was**……………………………………………………………………………………………………..................

No / Partly: What got in the way? ....

Physical Activity and Step-Count Diary **Week 6** **Daily Step-Count goal ________**

Weekly Step-Count goal _________

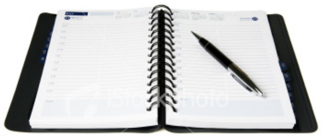

Use this diary to record any physical activities you do this week. This includes **walking** and **other physical activities** (e.g. cycling gardening, swimming, heavy housework, exercise classes etc). Write down the **approximate time** and **how long** you do them for.

**Date**							
**Day of week**							
**Morning**							
**Afternoon**							
**Evening**							
**Daily step-count**							

Add up the steps from all 7 days to get total weekly step-count: **Total weekly step-count = ________**

Divide the total weekly step-count by 7 to get average daily step-count: **Average daily step-count = ________**

Did I achieve my goal? **Yes: And my reward was**………………………………………………………………………………………….................

No / Partly: What got in the way? ....

Physical Activity and Step-Count Diary

Week 7 **Daily Step-Count goal ________**

Weekly Step-Count goal _________

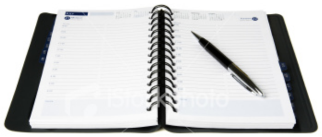

Use this diary to record any physical activities you do this week. This includes **walking** and **other physical activities** (e.g. cycling gardening, swimming, heavy housework, exercise classes etc). Write down the **approximate time** and **how long** you do them for.

**Date**							
**Day of week**							
**Morning**							
**Afternoon**							
**Evening**							
**Daily step-count**							

Add up the steps from all 7 days to get total weekly step-count: **Total weekly step-count = ________**

Divide the total weekly step-count by 7 to get average daily step-count: **Average daily step-count = ________**

Did I achieve my goal? **Yes: And my reward was**……

No / Partly: What got in the way? ……………………………………………………………………………………………………

Physical Activity and Step-Count Diary

Week 8 **Daily Step-Count goal ________**

Weekly Step-Count goal _________

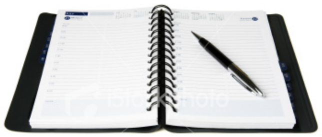

Use this diary to record any physical activities you do this week. This includes **walking** and **other physical activities** (e.g. cycling gardening, swimming, heavy housework, exercise classes etc). Write down the **approximate time** and **how long** you do them for.

**Date**							
**Day of week**							
**Morning**							
**Afternoon**							
**Evening**							
**Daily step-count**							

Add up the steps from all 7 days to get total weekly step-count: **Total weekly step-count = ________**

Divide the total weekly step-count by 7 to get average daily step-count: **Average daily step-count = ________**

Did I achieve my goal? **Yes: And my reward was**………………………………………………………………………………………………………

No / Partly: What got in the way? ………………………………………………………………………………………………

Physical Activity and Step-Count Diary **Week 9** **Daily Step-Count goal ________**

Weekly Step-Count goal _________

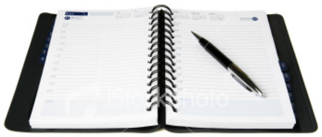

Use this diary to record any physical activities you do this week. This includes **walking** and **other physical activities** (e.g. cycling gardening, swimming, heavy housework, exercise classes etc). Write down the **approximate time** and **how long** you do them for.

**Date**							
**Day of week**							
**Morning**							
**Afternoon**							
**Evening**							
**Daily step-count**							

Add up the steps from all 7 days to get total weekly step-count: **Total weekly step-count = ________**

Divide the total weekly step-count by 7 to get average daily step-count: **Average daily step-count = ________**

Did I achieve my goal? **Yes: And my reward was**………

No / Partly: What got in the way? ……………………………………………………………………………………………………

Physical Activity and Step-Count Diary

Week 10 **Daily Step-Count goal ________**

Weekly Step-Count goal _________

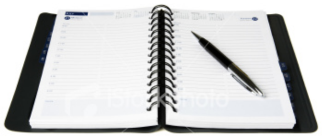

Use this diary to record any physical activities you do this week. This includes **walking** and **other physical activities** (e.g. cycling gardening, swimming, heavy housework, exercise classes etc). Write down the **approximate time** and **how long** you do them for.

**Date**							
**Day of week**							
**Morning**							
**Afternoon**							
**Evening**							
**Daily step-count**							

Add up the steps from all 7 days to get total weekly step-count: **Total weekly step-count = ________**

Divide the total weekly step-count by 7 to get average daily step-count: **Average daily step-count = ________**

Did I achieve my goal? **Yes: And my reward was**……........................

No / Partly: What got in the way? ....

Physical Activity and Step-Count Diary **Week 11** **Daily Step-Count goal ________**

Weekly Step-Count goal _________

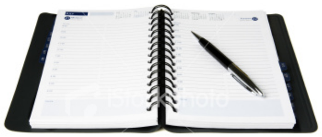

Use this diary to record any physical activities you do this week. This includes **walking** and **other physical activities** (e.g. cycling gardening, swimming, heavy housework, exercise classes etc). Write down the **approximate time** and **how long** you do them for.

**Date**							
**Day of week**							
**Morning**							
**Afternoon**							
**Evening**							
**Daily step-count**							

Add up the steps from all 7 days to get total weekly step-count: **Total weekly step-count = ________**

Divide the total weekly step-count by 7 to get average daily step-count: **Average daily step-count = ________**

Did I achieve my goal? **Yes: And my reward was**……………………………………………………………………………………………………

No / Partly: What got in the way? ……………………………………………………………………………………………

Physical Activity and Step-Count Diary **Week 12** **Daily Step-Count goal ________**

Weekly Step-Count goal _________

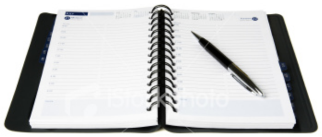

Use this diary to record any physical activities you do this week. This includes **walking** and **other physical activities** (e.g. cycling gardening, swimming, heavy housework, exercise classes etc). Write down the **approximate time** and **how long** you do them for.

**Date**							
**Day of week**							
**Morning**							
**Afternoon**							
**Evening**							
**Daily step-count**							

Add up the steps from all 7 days to get total weekly step-count: **Total weekly step-count = ________**

Divide the total weekly step-count by 7 to get average daily step-count: **Average daily step-count = ________**

Did I achieve my goal? **Yes: And my reward was**………..........................

No / Partly: What got in the way? ....

Physical Activity and Step-Count Diary

Week 13 **Daily Step-Count goal ________**

Weekly Step-Count goal _________

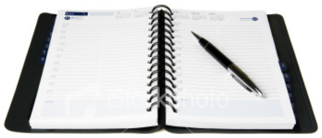

Use this diary to record any physical activities you do this week. This includes **walking** and **other physical activities** (e.g. cycling gardening, swimming, heavy housework, exercise classes etc). Write down the **approximate time** and **how long** you do them for.

**Date**							
**Day of week**							
**Morning**							
**Afternoon**							
**Evening**							
**Daily step-count**							

Add up the steps from all 7 days to get total weekly step-count: **Total weekly step-count = ________**

Divide the total weekly step-count by 7 to get average daily step-count: **Average daily step-count = ________**

Did I achieve my goal? **Yes: And my reward was**………

No / Partly: What got in the way? ……………………………………………………………………………………………………

Physical Activity and Step-Count Diary

Week

Daily Step-Count goal ________

Weekly Step-Count goal _________

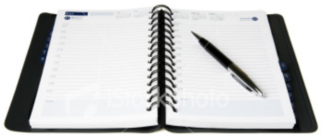

Use this diary to record any physical activities you do this week. This includes **walking** and **other physical activities** (e.g. cycling gardening, swimming, heavy housework, exercise classes etc). Write down the **approximate time** and **how long** you do them for.

**Date**							
**Day of week**							
**Morning**							
**Afternoon**							
**Evening**							
**Daily step-count**							

Add up the steps from all 7 days to get total weekly step-count: **Total weekly step-count = ________**

Divide the total weekly step-count by 7 to get average daily step-count: **Average daily step-count = ________**

Did I achieve my goal? **Yes: And my reward was**……………………………………………………………………………………………………

No / Partly: What got in the way? ……………………………………………………………………………………………

Physical Activity and Step-Count Diary

Week

Daily Step-Count goal ________

Weekly Step-Count goal _________

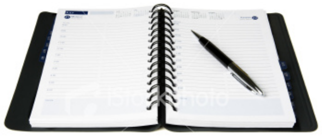

Use this diary to record any physical activities you do this week. This includes **walking** and **other physical activities** (e.g. cycling gardening, swimming, heavy housework, exercise classes etc). Write down the **approximate time** and **how long** you do them for.

**Date**							
**Day of week**							
**Morning**							
**Afternoon**							
**Evening**							
**Daily step-count**							

Add up the steps from all 7 days to get total weekly step-count: **Total weekly step-count = ________**

Divide the total weekly step-count by 7 to get average daily step-count: **Average daily step-count = ________**

Did I achieve my goal? **Yes: And my reward was**………

No / Partly: What got in the way? ……………………………………………………………………………………………………

Physical Activity and Step-Count Diary **Week** **Daily Step-Count goal ________**

Weekly Step-Count goal _________

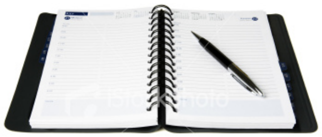

Use this diary to record any physical activities you do this week. This includes **walking** and **other physical activities** (e.g. cycling gardening, swimming, heavy housework, exercise classes etc). Write down the **approximate time** and **how long** you do them for.

**Date**							
**Day of week**							
**Morning**							
**Afternoon**							
**Evening**							
**Daily step-count**							

Add up the steps from all 7 days to get total weekly step-count: **Total weekly step-count = ________**

Divide the total weekly step-count by 7 to get average daily step-count: **Average daily step-count = ________**

Did I achieve my goal? **Yes: And my reward was**...

No / Partly: What got in the way? ....
