# Supplementary material for: A Primary Care Nurse-Delivered Walking Intervention in Older Adults: PACE (Pedometer Accelerometer Consultation Evaluation)-Lift Cluster Randomised Controlled Trial
Source: PLoS Med. 2015 Feb 17;12(2):e1001783. doi: 10.1371/journal.pmed.1001783 (PMC4331517; doi:10.1371/journal.pmed.1001783)
Supplement: S5 Text — (DOCX) [file pmed.1001783.s011.docx]

**Text S5: Estimating reduction in risk of coronary heart disease and type 2 diabetes**

Several systematic reviews have assessed the benefits of walking based on pooling data from cohort studies. Typically the relative risks are 0.8 in those who are physically more active compared to those who are much less active. The difficulty of interpreting such analyses is their focus on comparing two extreme groups; the physically active vs those inactive. Zheng et al [34] in their review recognised the importance of studying the functional form of the dose response effect of walking on coronary heart disease (CHD) risk. They concluded that the risk of CHD decreases steadily as amount of brisk walking increases. Specifically they concluded that 150 minutes of brisk walking/week reduces CHD by 19% (a RR of 0.81 (95%CI 0.77, 0.86); relative risk estimates were similar in both sexes and in older and younger subjects. Based on the findings of Zheng et al that log (Risk) increases linearly with minutes of MVPA we can estimate that increasing MVPA by 40 minutes/week results in a relative risk of 0.81^(40/150)^ = 0.81^0.267^ = 0.945. That is a reduction in risk of CHD of 5.5% (95%CI 3.9%, 6.7%) if the 40 minutes difference in MVPA were sustained.

Similarly in another meta-analysis, Jeon et al [35] concluded that the risk of type 2 diabetes in those walking regularly (typically >=150 minutes /week of brisk walking) was reduced by 30% (RR 0.70 (95%CI 0.58-0.84)) compared to those with almost no walking; again several of the individual studies included demonstrated clear dose response effects and the reduced risk of type 2 diabetes was only partially explained by adjustment for BMI, the RR being 0.83 (0.75-0.91) after adjustment. [35]. Making the same assumption as for CHD we estimate that in our study, increasing MVPA by 40 minutes would be associated with a relative risk of by 0.70^(40/150)^ = 0.70^0.267^ = 0.91. That is the risk of type 2 diabetes would be reduced by 9.1% (4.5%-13.5%), if the 40 minute difference were sustained.
